# imputomics: web server and R package for missing values imputation in metabolomics data

**DOI:** 10.1093/bioinformatics/btae098

**Published:** 2024-02-20

**Authors:** Jarosław Chilimoniuk, Krystyna Grzesiak, Jakub Kała, Dominik Nowakowski, Adam Krętowski, Rafał Kolenda, Michał Ciborowski, Michał Burdukiewicz

**Affiliations:** Clinical Research Centre, Medical University of Białystok, Białystok, Poland; Clinical Research Centre, Medical University of Białystok, Białystok, Poland; Faculty of Mathematics and Computer Science, University of Wrocław, Wrocław, Poland; Clinical Research Centre, Medical University of Białystok, Białystok, Poland; Department of Biostatistics and Medical Informatics, Medical University of Białystok, Białystok, Poland; Clinical Research Centre, Medical University of Białystok, Białystok, Poland; Quadram Institute Biosciences, Norwich Research Park, Norwich, United Kingdom; Faculty of Veterinary Medicine, Wrocław University of Environmental and Life Sciences, Wrocław, Poland; Clinical Research Centre, Medical University of Białystok, Białystok, Poland; Clinical Research Centre, Medical University of Białystok, Białystok, Poland; Institute of Biotechnology and Biomedicine, Autonomous University of Barcelona, Cerdanyola del Vallès, Spain

## Abstract

**Motivation:**

Missing values are commonly observed in metabolomics data from mass spectrometry. Imputing them is crucial because it assures data completeness, increases the statistical power of analyses, prevents inaccurate results, and improves the quality of exploratory analysis, statistical modeling, and machine learning. Numerous Missing Value Imputation Algorithms (MVIAs) employ heuristics or statistical models to replace missing information with estimates. In the context of metabolomics data, we identified 52 MVIAs implemented across 70 R functions. Nevertheless, the usage of those 52 established methods poses challenges due to package dependency issues, lack of documentation, and their instability.

**Results:**

Our R package, ‘imputomics’, provides a convenient wrapper around 41 (plus random imputation as a baseline model) out of 52 MVIAs in the form of a command-line tool and a web application. In addition, we propose a novel functionality for selecting MVIAs recommended for metabolomics data with the best performance or execution time.

**Availability and implementation:**

‘imputomics’ is freely available as an R package (github.com/BioGenies/imputomics) and a Shiny web application (biogenies.info/imputomics-ws). The documentation is available at biogenies.info/imputomics.

## 1 Introduction

Metabolomics is a quickly growing branch of ‘-omics’ studies that focus on the detection of small molecules, known as metabolites, produced by living organisms and biological systems on different levels of the organization. By studying them, researchers can acquire insight into the organism’s phenotype affected by diverse factors such as genetics or diet. Hence, metabolomics has numerous applications, starting with medicine, food sciences, and ending with environmental biology.

Metabolite detection involves the usage of cutting-edge analytical techniques, such as mass spectrometry (MS) paired with gas or liquid chromatography (Villas-Bôas *et al.* 2005, Zampieri *et al.* 2017). Due to the sheer variability of metabolites, sensitivity of analytical instruments, and high-throughput nature of those techniques, enormous amounts of data are generated. However, technical and biological factors like poor sample quality or instrumental limitations cause the appearance of missing values, hindering subsequent data analysis (Armitage *et al.* 2015).

Ignoring missing values is problematic because it leads to the loss of valuable information and can result in erroneous conclusions, ultimately decreasing the credibility and utility of data analysis. However, addressing missing values may be equally problematic, as each type of missing data requires specific consideration and handling methods, such as data imputation. In general, there are three types of missing value patterns. Missing completely at random (MCAR), when missing values are due to random errors and stochastic fluctuations during the data acquisition process, such as incomplete derivation or ionization. Missing at random (MAR) assumes that other observed variables determine the possibility of missing a variable. Missing not at random (MNAR), is considered for censored missing values, that are caused by the limit of detection (LOD) of a device (Rubin 1976, Karpievitch *et al.* 2012). In most real-world scenarios, it is expected to encounter mixtures of at least two types of missing data patterns.

The importance of this issue led the metabolomics community to use existing or introduce new missing value imputation algorithms (MVIAs), which replace the missing value with its estimate. Our extensive literature review (Supplementary data SI1) revealed that 52 MVIAs proposed for metabolomics data are implemented in 70 R functions (Supplementary data SI2) and described in 20 articles. Moreover, the most robust web server for missing data imputation in metabolomics, MetImp (Wei *et al.* 2018), covers only 10 out of 52 proposed MVIAs (19.23%).

Nonetheless, such a wide range of diverse methods imposes new challenges. The maintenance level and implementation quality of available MVIAs differs from algorithm to algorithm, leading to problems with usage, stability, and reproducibility. The latter is also affected by the lack of common standards (e.g. the presence of hard-coded random seeds). Moreover, the usage of some MVIAs is hindered by their specific environment requirements that are hard to reproduce by less R-fluent users.

In response to the complexities introduced by the multitude of MVIAs and the challenges they pose, we recognized the need for a streamlined and user-friendly solution. We have developed an R package ‘imputomics’, which contains a comprehensive set of imputation methods while providing a unified and intuitive programming interface. It aims to simplify the imputation process for researchers by reducing the burden of managing package dependencies, enhance code maintainability, and help in selecting appropriate MVIAs. Furthermore, to make data imputation more accessible, we have established an ‘imputomics’ web application. It facilitates not only missingness analysis, data imputation, and diagnosis of the imputed data but also allows users to leverage insights from our benchmark comparing the performance of MVIAs. Based on the benchmark results, users can confidently choose the best-performing or fastest methods that align with their specific research needs.

## 2 Implementation

First, we have gathered a collection of articles related to MVIAs in metabolomics research (collection, curation, and extraction pipeline can be seen in Supplementary Fig. S1). We have selected the MVIAs written in R and used for metabolomics data imputation. We have also added (with permission of the authors) two MVIAs that were already proposed but never shared publicly (Shah *et al.* 2019). In addition to that, we brought back to CRAN implementations of MVIAs that were removed due to the lack of maintenance (Perry *et al.* 2023). Additionally, we have implemented random imputation (imputation using random numbers within the variable’s range) as a baseline for performance comparison with other MVIAs. Finally, ‘imputomics’ package contains 41 (plus random imputation as a baseline MVIA) out of the 52 identified MVIAs. We excluded a total of 11 methods due to at least one of the following reasons:

- The code does not work without significant alterations, even for default data.
- MVIA requires additional information such as spectrum peaks or class labels.
- The exact package or function is not specified in the associated research paper.
- At least one of the columns has to contain no missing values.
- Besides imputation, MVIA modifies non-missing data points.

The list of removed methods, citations, and references to the code can be found in Supplementary data SI1.1. This approach ensures that all methods in the ‘imputomics’ package have the same scope and application.

One of the main goals of our study was also to enhance the reproducibility of MVIAs. Thus, we have created a stable environment for computations using the ‘renv’ R package (Ushey and Wickham 2023), which isolates project dependencies, minimizing the risk of conflicts and ensuring consistency. Moreover, for computational safety and convenience, we have implemented individualized wrapper functions for each MVIAs. Each wrapper is tailored to perform the following tasks:

1. Uniformity: The wrapper function ensures that all imputing functions accept and return ‘data.frames’ (and, by extension, ‘tibbles’) to ensure the tidy data processing (Wickham *et al.* 2019). We also revert all other changes introduced by the original imputing functions irrelevant to the missing data imputation, such as the change of column names or transposition of original data. Moreover, we designed all helper functions (e.g. scaling) only to accept ‘data.frames’.
2. Input data verification: Our wrapper functions automatically check for the presence of missing values in the form of ‘Not Available’ (NAs) or zeros/ones within the data (these numbers are commonly used in metabolomics to denote missing values). We also ensure that the provided input data consists exclusively of numeric, non-negative values. In addition, we perform additional data integrity checks if the authors of MVIA advise them but do not implement them in their original function.
3. Passing arguments to function calls: The wrapper ensures that relevant arguments are appropriately passed to the underlying imputation functions while keeping the default values from the original implementations. If we alter the default behavior of the function (e.g. by removing a predefined random seed), this information is marked in the documentation. To prevent unnecessary screen prompts produced during the imputation, each function is additionally enhanced with the boolean ‘verbose’ argument.

To facilitate evaluation and testing of imputing functions, ‘imputomics’ also possesses amputation functions allowing building synthetic datasets containing missing MCAR, MAR, and MNAR values as well as their mixtures. These functions were tailored to simulate real-life situations occurring in metabolomics data as LODs (Supplementary data SI3.2).

**Figure 1.**
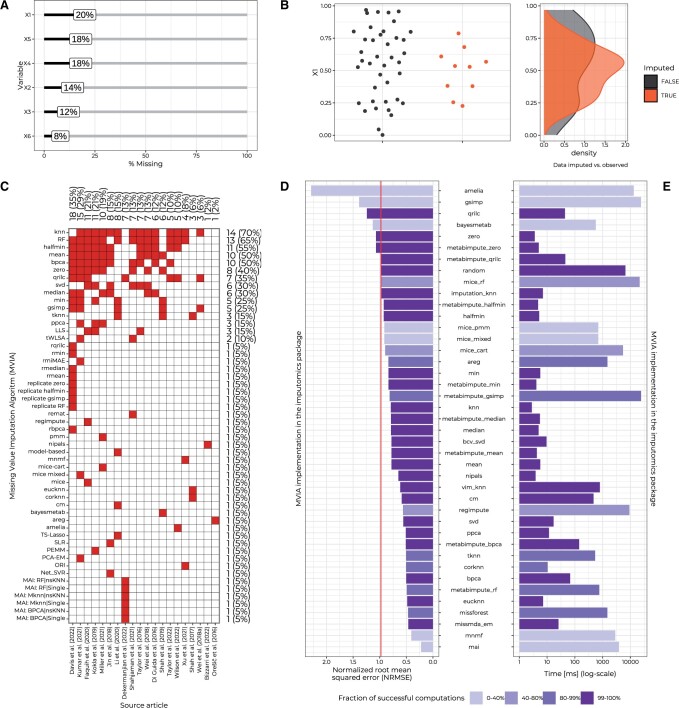
(A) A graphical representation of missing values in a preliminary analysis of dataset. (B) Distribution of imputed data compared to the observed data. (C) Occurrence of MVIAs (missing value imputation algorithms). Filled squares mark the presence of a given MVIA. The right-hand side annotations represent the number of MVIAs covered by a given article. The top annotations represent the articles covering a given MVIA. (D) Normalized root mean squared error (NRMSE) of MVIAs. The vertical line marks the baseline MVIA: random imputation. As the NRMSE for PEMM exceeded $6.03\times 10^{16}$, this MVIA is not represented on the chart. (E) The maximum time [ms] necessary to impute missing values. Both in (D) and (E), the color of the bars marks the percentage of datasets on which an MVIA converged successfully in under 2 min.

Profiting from having a robust wrapper over MVIAs, we created a user-friendly web application based on the shiny framework (Chang *et al.* 2024). Our tool enables less R-fluent researchers to impute missing values in their datasets. In addition, it provides graphical methods to examine the input data (Fig. 1A) and inspect the distribution of imputed data (explained in Supplementary data SI8) (Fig. 1B). The ‘imputomics’ shiny application is available as a web server (biogenies.info/imputomics-ws). The R package and its detailed installation guide can be found at github.com/BioGenies/imputomics.

To further guide users of our web application, we attempted to determine which methods deliver the most accurate imputation or exhibit the highest speed based on existing literature. However, there is no definitive answer to that question. The most extensive benchmark of MVIAs for assessing the accuracy, execution time, and computational stability (Davis *et al.* 2022) covers only 18 algorithms, which is 34.62% of all considered MVIAs (Fig. 1C) and 25.71% of all considered implementations (Supplementary data SI2 and Supplementary Fig. S2). Therefore, to assist users in choosing the most suitable MVIA from a wide array of available methods, we have decided to conduct a benchmark to showcase their performance.

To evaluate the performance of MVIAs, we ran them with their default parameters in all 230 scenarios (10 missing values patterns × 23 different-sized datasets). During our preliminary assessment, we noticed that MVIAs either converged quite fast or were imputing data endlessly, so we decided to limit the computation time to two minutes per single run of the MVIA code. Each MVIA was executed three times, if the attempt failed to impute MV in each of the three approaches, we marked it as not working for a given scenario (Supplementary data SI3.3). To assess the imputation quality, we utilized the normalized root mean squared error (NRMSE). The NRMSE is a widely accepted measure for quantifying the accuracy of imputed data, as it is not sensitive to heteroscedasticity among the variables, allowing for relative error assessment (Supplementary data SI4) (Oba *et al.* 2003).

Furthermore, to assist users of our web server in selecting the most suitable method for their dataset, we introduced the option to choose methods that demonstrated the best performance in simulations—in terms of speed, overall effectiveness, and suitability for specific missing value patterns (i.e. MCAR, MAR, and MNAR).

## 3 Results and conclusions

‘imputomics’ stands as the most comprehensive R package for the imputation of missing data in R, encompassing 42 MVIAs. It represents an effort to bridge the gap between the richness of available methods and the practical usability of these techniques, making advanced data imputation accessible to a broader spectrum of researchers in the field of metabolomics.

The instant accessibility of multiple MVIAs enables the choice of MVIAs based on the covariate distribution and the missing value pattern. However, we warn potential users against selecting the method to support a preconceived hypothesis. The choice of the optimal MVIA should happen *a priori* or result from an understanding of the data structure. To guide such choices, our simulation results provide a more comprehensive understanding of the performance of various MVIA implementations depending on the type of missingness and the data dimensionality.

Out of 42 MVIA implementations, 17 (40.48%) would not consistently succeed in every scenario (Fig. 1D). It is important to emphasize that 25.53% of errors are attributed to instances where convergence failed to occur within the defined timeout. This instability can sometimes be attributed to running MVIAs implementations with default parameters. However, this outcome holds significance for users who do not intend to fine-tune their missing value imputation pipeline or seek a robust solution that offers relatively swift imputation. Among the most stable algorithms, the top performers include EM from the R package missMDA (Josse and Husson 2016), and KNN-EU (Shah *et al.* 2019), both achieving NRMSE values lower than 0.5. Eight MVIAs implementations (19.05%) failed to surpass the baseline model, which, in our case, involves imputation by a random number (Fig. 1D and Supplementary data SI5). Depending on the type of missing data (MCAR, MAR, and, resulting from LOD, MNAR), different MVIAs respectively had the best performance: Random Forest (as implemented in MetabImpute) (Davis *et al.* 2022), missMDA (Josse and Husson 2016), and min (as implemented in MetabImpute or ‘imputomics’ packages) (Supplementary data SI6).

The benchmarked MVIAs exhibit considerable diversity in their evaluation times (Fig. 1E and Supplementary data SI5). Interestingly, there is no significant correlation between the performance of MVIAs implementations and their computation time (*P*-value: 0.13, Spearman’s correlation coefficient: −0.31).

Looking ahead, we anticipate ‘imputomics’ to streamline and tidy up the complicated landscape of the missing value imputation in R. We consider metabolomics as an appropriate starting point to extend our package to cover also MVIAs proposed for other branches of ‘-omics’. Presently, both ‘imputomics’ R package and web application for the first time provide easy and robust access to the majority of MVIAs implemented in R.

## Supplementary Material

btae098_Supplementary_Data

## Data Availability

The data underlying this article are available in the article and in its online supplementary material.
