# Supplementary material for: imputomics: web server and R package for missing values imputation in metabolomics data
Source: Bioinformatics. 2024 Feb 20;40(3):btae098. doi: 10.1093/bioinformatics/btae098 (PMC10918629; doi:10.1093/bioinformatics/btae098)
Supplement: btae098_Supplementary_Data [file btae098_supplementary_data.pdf]

Supplementary Information

imputomics: web server and R package for missing values imputation in metabolomics data

Jarosław Chilimoniuk [1,†], Krystyna Grzesiak [2, 1,†], Jakub Kała [1], Dominik Nowakowski [3], Adam Krętowski [1], Rafał Kolenda [4, 5], Michał Ciborowski [1], Michał Burdukiewicz [6, 1,*]

[1]Clinical Research Centre, Medical University of Białystok, Poland [2]Faculty of Mathematics and Computer Science, University of Wrocław, Poland [3]Department of Biostatistics and Medical Informatics, Medical University of Białystok, Poland [4]Quadram Institute Biosciences, Norwich Research Park, Norwich, UK [5]Wrocław University of Environmental and Life Sciences, Poland [6]Institute of Biotechnology and Biomedicine, Autonomous University of Barcelona, Cerdanyola del Vallès, Spain.

Contents

1 Acquisition of missing value imputation algorithms **3**
1.1 Exclusion of MVIAs 3

2 MVIAs **5**

3 Missing value scenarios **10**
3.1 Obtaining datasets from MW - datasets preparation 10
3.2 Amputation 11
3.2.1 MCAR 12
3.2.2 MAR 12
3.2.3 MNAR 13
3.2.4 Mixtures 13
3.3 Imputation 13

4 Performance measure: NRMSE **15**

5 Results of simulation **16**

6 Summary of simulation results **25**

7 Example uses of imputomics **27**

8 imputomics web server **29**

9 References **35**

SI1 Acquisition of missing value imputation algorithms

We gathered a collection of articles related to missing value imputation algorithms (MVIAs) in metabolomics research were gathered using the following PubMed:

- "imputation missing metabolomics"
- "metabolomics missing values",
- "metabolomics missing"
- "metabolomics imputation"

We checked if found publications used metabolomics data for the imputation. Then we selected only those MVIAs that were written in R (R Core Team 2022). The full pipeline of collection, curation, and extraction of imputation functions from the articles can be seen in Figure S1.

Our search yielded 26 articles that employed various methods for imputing missing values in metabolomics datasets. 20 of them (76.92%) employed at least one method implemented in R (R Core Team 2022). Within those articles, we identified 52 distinct MVIAs with 70 different implementations.

As a baseline model, we have added random imputation, where a missing value in a column is imputed with a randomly sampled non-missing value from the column. We assume that if every MVIA that fails to outperform the random imputation should be applied very carefully.

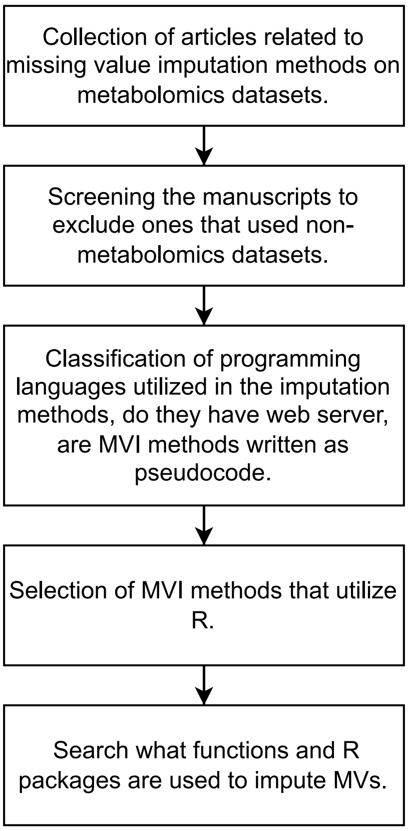

Figure S1: **Scheme for gathering and curating articles.**

As the code necessary to use certain MVIAs (such as MINMA, BayesMetab, or ORI) was not readily available, we have emailed the respective authors to obtain access to them. We received access to MINMA and BayesMetab from their respective authors.

SI1.1 Exclusion of MVIAs

We have chosen MVIAs to implement using the following criteria:

1. Runs on default parameters without requiring additional data besides metabolite intensities.
2. Each column can contain missing values.
3. Does not modify original data besides imputing missing values.

Thus, several imputation methods were excluded from our analysis as they didn't meet our standards. The kNN-obs-sel and MICE-pmm methods from the UnMetImp package and impute.SLR function from MINMA were eliminated, as we were unable to run them on default hyperparameters as they need more than metabolites intensities. Similarly,

llsImpute from pcaMethods, rfImpute from randomForest, TS.Lasso from GSMimpute, and MINMA from MINMA were removed because they required at least one column to be free of missing values. The MBimpute function from DanteR and remat from rMisbeta were excluded as they modify the data during the imputation process.

We chose not to include the random forest, kNN-TN, and kNN imputation methods employed in the study published in (Li et al. 2020), as the specific implementations used were not specified. Furthermore, we were unable to obtain the ORI code from the authors despite our request for access.

In terms of the MAI package, we only incorporated the best-performing imputation methods for missing value types ('Random forest' as the MCAR algorithm and 'Single' as the MNAR algorithm).

SI2 MVIAs

Table S1: **List of all found MVIAs implemented in R along with their corresponding implementations and articles where they were used.** The *full name* column indicates the name of the MVIA. The *implementation* column contains the R package and function used, the square brackets indicate the package name. If no package is listed, the imputation function was taken from the paper supplements. If there wasn't a specified implementation of MVIA then it is annotated as unknown. The 'base' label indicates functions implemented in base R. The *citation* column lists the papers where the method was used.

full name	implementation	citation
half-minimum imputation	[base]	Di Guida et al. (2016); Miller et al. (2021); Wei et al. (2018); Kumar, Hoque, and Sugimoto (2021); S. Taylor et al. (2022); S. L. Taylor et al. (2016); Jin, Kang, and Yu (2018); Kokla et al. (2019); Faquih et al. (2020); Wilson et al. (2022)
mean imputation	[base]	Di Guida et al. (2016); Miller et al. (2021); Wei et al. (2018); Kumar, Hoque, and Sugimoto (2021); S. L. Taylor et al. (2016); Jin, Kang, and Yu (2018); Kokla et al. (2019); J. Shah, Brock, and Gaskins (2019); Faquih et al. (2020)
median imputation	[base]	Di Guida et al. (2016); Miller et al. (2021); Wei et al. (2018); Kumar, Hoque, and Sugimoto (2021); Jin, Kang, and Yu (2018)
minimum imputation	[base]	Kumar, Hoque, and Sugimoto (2021); Li et al. (2020); Kokla et al. (2019); J. Shah, Brock, and Gaskins (2019)
zero imputation	[base]	Miller et al. (2021); Shahjaman et al. (2021); Wei et al. (2018); Kumar, Hoque, and Sugimoto (2021); Kokla et al. (2019); J. Shah, Brock, and Gaskins (2019); Faquih et al. (2020)
Amelia: bootstrap EM	amelia [Amelia]	Wilson et al. (2022)
multiple imputation additive regression	aregImpute [Hmisc]	Orešič et al. (2016)
glmnet ridge regression	DreamAI(method = "RegImpute") [DreamAI]	Faquih et al. (2020)
compound minimium	GMS.Lasso(TS.Lasso = FALSE) [GMSimpute]	Li et al. (2020)
Gibbs sampler imputation	GS_impute [GSimp]	Kumar, Hoque, and Sugimoto (2021); Li et al. (2020); J. Shah, Brock, and Gaskins (2019); Wei et al. (2018a)
Multiple Imputation by Chained Equations Mixed	impute_data(sel_method = 11) [missCompare]	Kumar, Hoque, and Sugimoto (2021)
k-nearest neighbors	impute.knn [impute]	Di Guida et al. (2016); Shahjaman et al. (2021); Wei et al. (2018); Kumar, Hoque, and Sugimoto (2021); S. Taylor et al. (2022); S. L. Taylor et al. (2016); Jin, Kang, and Yu (2018); Kokla et al. (2019); Wei et al. (2018a); Wilson et al. (2022); Xu et al. (2021)
quantile regression approach for the imputation of left-censored	impute.QRILC [imputeLCMD]	Kumar, Hoque, and Sugimoto (2021); S. Taylor et al. (2022); Dekermanjian et al. (2022); Wei et al. (2018a); Faquih et al. (2020); Wilson et al. (2022)
Simple Linear Regression	impute.SLR [MINMA]	Jin, Kang, and Yu (2018)
Singular Value Decomposition	impute.svd [bcv]	Jin, Kang, and Yu (2018); Faquih et al. (2020)
Gibbs sampler imputation	Impute(method == 'GSIMP') [MetabImpute]	Davis et al. (2022)
mean imputation	Impute(method == 'mean') [MetabImpute]	Davis et al. (2022)
median imputation	Impute(method == 'median') [MetabImpute]	Davis et al. (2022)

full name	implementation	citation
minimum imputation	Impute(method == 'min') [MetabImpute]	Davis et al. (2022)
quantile regression approach for the imputation of left-censored	Impute(method == 'QRILC') [MetabImpute]	Davis et al. (2022)
replicate Bayesian Principal Component Analysis	Impute(method == 'RBPCA') [MetabImpute]	Davis et al. (2022)
Random Forest	Impute(method == 'RF') [MetabImpute]	Davis et al. (2022)
replicate half-minimum imputation	Impute(method == 'RHM') [MetabImpute]	Davis et al. (2022)
zero imputation	Impute(method == 'zero') [MetabImpute]	Davis et al. (2022)
Bayesian Principal Component Analysis	Impute(method == "BPCA") [MetabImpute]	Davis et al. (2022)
half-minimum imputation	Impute(method == "halfmin") [MetabImpute]	Davis et al. (2022)
replicate Gibbs sampler imputation	Impute(method == "RGSIMP") [MetabImpute]	Davis et al. (2022)
replicate mean imputation	Impute(method == "RMEAN") [MetabImpute]	Davis et al. (2022)
replicate median imputation	Impute(method == "RMEDIAN") [MetabImpute]	Davis et al. (2022)
replicate minimum imputation	Impute(method == "RMIN") [MetabImpute]	Davis et al. (2022)
replicate quantile regression approach for the imputation of left-censored	Impute(method == "RQRILC") [MetabImpute]	Davis et al. (2022)
Replicate Random Forest	Impute(method == "RRF") [MetabImpute]	Davis et al. (2022)
replicate zero imputation	Impute(method == "RZERO") [MetabImpute]	Davis et al. (2022)
k-nearest neighbors correlation	imputeKNN(distance = "correlation")	J. S. Shah et al. (2017)
K-nearest neighbor truncation	imputeKNN(distance = "truncation")	Li et al. (2020); J. S. Shah et al. (2017); J. Shah, Brock, and Gaskins (2019)
iterative PCA	imputePCA(method = "EM") [missMDA]	Kumar, Hoque, and Sugimoto (2021)
k-nearest neighbors	knn [VIM]	Miller et al. (2021)
k-nearest neighbors euclidean	KNNEuc	J. S. Shah et al. (2017)
k-nearest neighbors	kNNImpute imputation	Faquih et al. (2020)
Local Least Squares	llsImpute [pcaMethods]	S. L. Taylor et al. (2016); Kokla et al. (2019); Faquih et al. (2020)
Mechanism-Aware Imputation: BPCA & nsKNN	MAI(MCAR_algorithm = "BPCA", MNAR_algorithm = "nsKNN")) [MAI]	Dekermanjian et al. (2022)

full name	implementation	citation
Mechanism-Aware Imputation: BPCA & Single	MAI(MCAR_algorithm = “BPCA”, MNAR_algorithm = “Single”) [MAI]	Dekermanjian et al. (2022)
Mechanism-Aware Imputation: Multi_nsKNN & Single	MAI(MCAR_algorithm = “Multi_nsKNN”, MNAR_algorithm = “Single”) [MAI]	Dekermanjian et al. (2022)
Mechanism-Aware Imputation: Multi_nsKNN & nsKNN	MAI(MCAR_algorithm = “Multi_nsKNN”, MNAR_algorithm = “nsKNN”) [MAI]	Dekermanjian et al. (2022)
Mechanism-Aware Imputation: random_forest & Single	MAI(MCAR_algorithm = “random_forest”, MNAR_algorithm = “Single”) [MAI]	Dekermanjian et al. (2022)
Mechanism-Aware Imputation: random_forest & nsKNN	MAI(MCAR_algorithm = “random_forest”, MNAR_algorithm = “nsKNN”) [MAI]	Dekermanjian et al. (2022)
Model-based imputation	MBimpute() [DanteR]	Li et al. (2020)
BayesMetab	MCMC.Factor [BayesMetab]	J. Shah, Brock, and Gaskins (2019)
Classification And Regression Trees	mice(method = “cart”) [mice]	Miller et al. (2021)
Predictive Mean Matching	mice(method = “pmm”) [mice]	Miller et al. (2021)
Random Forest	mice(method = “rf”) [mice]	Miller et al. (2021)
Feature-level predictor network SVR model imputation	MINMA [MINMA]	Jin, Kang, and Yu (2018)
Random Forest	missForest [missForest]	Di Guida et al. (2016); Shahjaman et al. (2021); Wei et al. (2018); Kumar, Hoque, and Sugimoto (2021); S. Taylor et al. (2022); S. L. Taylor et al. (2016); Kokla et al. (2019); Faquih et al. (2020); Wilson et al. (2022); Xu et al. (2021)
Non-negative Matrix Factorization	nmf_opt [NMF]	Xu et al. (2021)
Bayesian Principal Component Analysis	pca(method = “bpca”) [pcaMethods]	Di Guida et al. (2016); Miller et al. (2021); Shahjaman et al. (2021); Kumar, Hoque, and Sugimoto (2021); S. Taylor et al. (2022); S. L. Taylor et al. (2016); Jin, Kang, and Yu (2018); Kokla et al. (2019); Faquih et al. (2020)
Non-Linear Iterative Partial Least Squares	pca(method = “nipals”) [pcaMethods]	Bizzarri et al. (2022)
Probabilistic Principal Component Analysis	pca(method = “ppca”) [pcaMethods]	Miller et al. (2021); Kumar, Hoque, and Sugimoto (2021); Kokla et al. (2019)
Singular Value Decomposition	pca(method = “svdImpute”) [pcaMethods]	Shahjaman et al. (2021); Wei et al. (2018); S. L. Taylor et al. (2016); Kokla et al. (2019)
Penalized Expectation Maximization	PEMM_fun [PEMM]	Kokla et al. (2019)
robust estimators minimum beta divergence method	remat [rMisbeta]	Shahjaman et al. (2021)
Random Forest	rfImpute [randomForest]	Shahjaman et al. (2021)

full name	implementation	citation
Robust Missing imputation by minimizing two way mean absolute error	rmiMAE [rmiMAE]	Kumar, Hoque, and Sugimoto (2021)
Two-Step LASSO	TS.Lasso [GMSimpute]	Li et al. (2020)
K-nearest neighbor truncation	[unknown]	Li et al. (2020)
k-nearest neighbors	[unknown]	Li et al. (2020)
Outlier-Robust Imputation	[unknown]	Xu et al. (2021)
Random Forest	[unknown]	Li et al. (2020)
k-nearest neighbors	UnMetImp(imp_type = “knn”) [UnMetImp]	Faquih et al. (2020)
Multiple Imputation by Chained Equations	UnMetImp(imp_type = “mice”) [UnMetImp]	Faquih et al. (2020)
Two way Kernel Weighted Least Square Approach	wlsMisImp [tWLSA]	Shahjaman et al. (2021); Kumar, Hoque, and Sugimoto (2021)

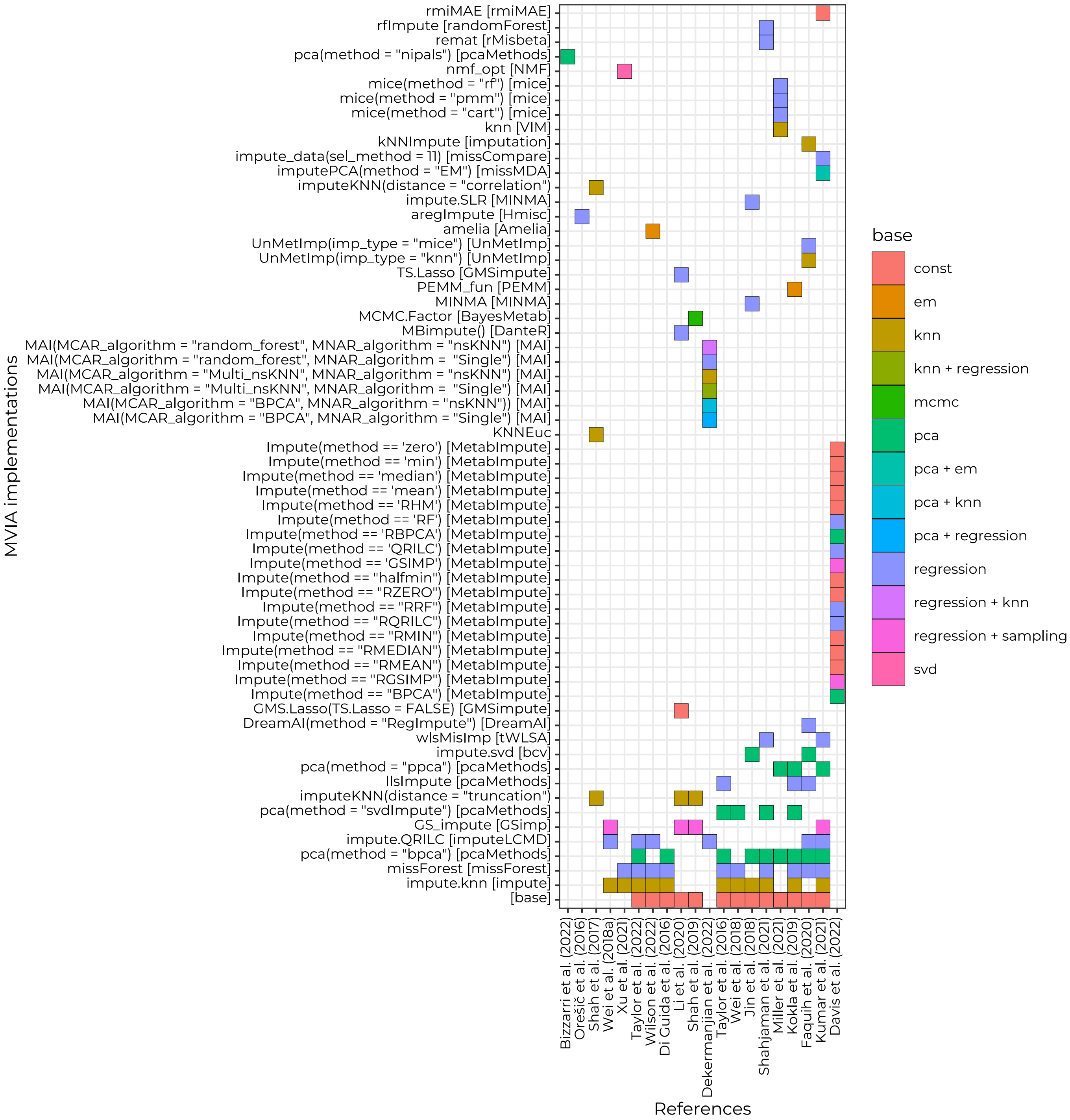

Figure S2: **MVIA used on metabolomics datasets.** Tile plot shows on Y-axis MVIAs implementations and in square brackets R packages from which they originate. The imputation function was taken from the paper supplements if no package is listed. The 'base' label indicates functions implemented in base R, e.g. mean, median, zero. The X-axis shows articles where corresponding implementations were used. The unknown implementations were removed.

SI3 Missing value scenarios

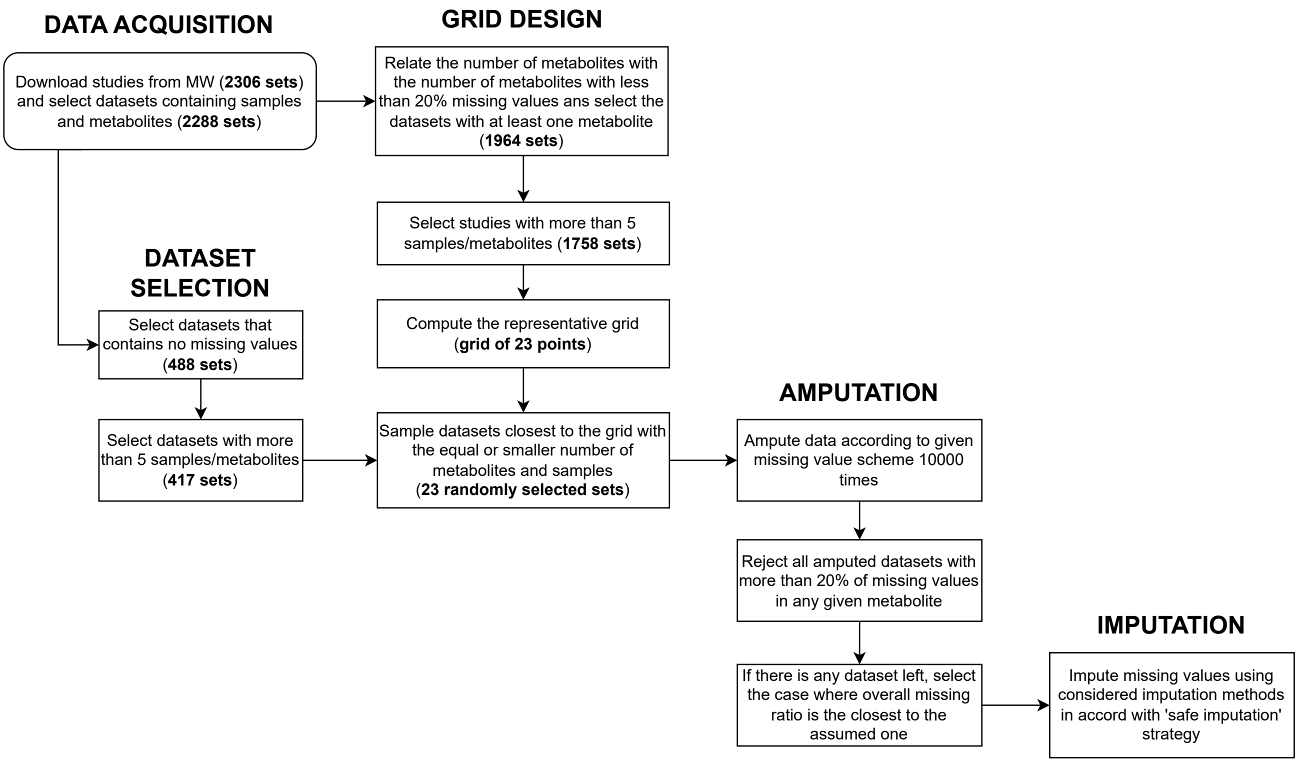

Figure S3: **Scheme of the dataset selection, data amputation and MVIAs benchmark.**

SI3.1 Obtaining datasets from MW - datasets preparation

On March 10, 2023, we employed HTTP requests through the REST API to retrieve 2,306 sets of data from Metabolomics Workbench (MW). Subsequently, the datasets lacking numeric values and consisting solely of named samples with named groups were excluded. These datasets represent much larger (sample- or metabolite-wise) studies that we have excluded due to the computational limitations on our side. This resulted in a collection of 2,288 datasets of metabolite intensities (Fig. S3, **DATA ACQUISITION**).

Next, we defined the number of metabolites in a dataset as the number of metabolites that have no more than 40% missing values (denoted as zeros in MW), and we selected those studies that have at least 5 metabolites/samples, which resulted in a total of 1812 datasets. Then, referring to the marginal distributions of numbers of metabolites/samples, we created a grid comprising 23 points (6 compounds $\times$ 4 samples - 1). The compounds spanned a range from 10 to 370, while the samples varied from 10 to 195 (Fig. S4). This grid represents the most commonly occurring relations between sample number and metabolite number in MW (Fig. S3, **GRID DESIGN**).

Next, we were looking for datasets without missing values to find ones suitable for the benchmark of imputation methods. Thus, we identified 488 datasets without zeroes in MW studies (representing missing values, which was confirmed by personal correspondence with the MW support), out of which 417 contained at least 5 samples/metabolites. Afterward, we calculated the *distance* of each dataset from the points within our grid. In this context, *distance* is defined as the joint difference between the number of metabolites/samples present in the dataset and the corresponding number of metabolites/samples for a given point in the grid. Based on that measure, we identified the datasets that are the closest to the points from our grid. If more than one dataset was at the same *distance* from the grid point, we have randomly sampled one of them (Fig. S3, **DATASET SELECTION**). The 23 selected datasets (and their *distance* from the specific grid point) are presented in Table S2. The selected datasets are also presented visually in Figure S5.

```
library(xtable)
grid_datasets <- readRDS("./data/grid_datasets.RDS")

table_string <- grid_datasets %>%
  select(name, actual_smp, smp, smp_diff,
         actual_cmp, cmp, cmp_diff, total_diff) %>%
  rename(cmp_grid = cmp, smp_grid = smp) %>%
  rename(cmp = actual_cmp, smp = actual_smp) %>%
  mutate_if(is.numeric, as.integer) %>%
  xtable() %>%
```

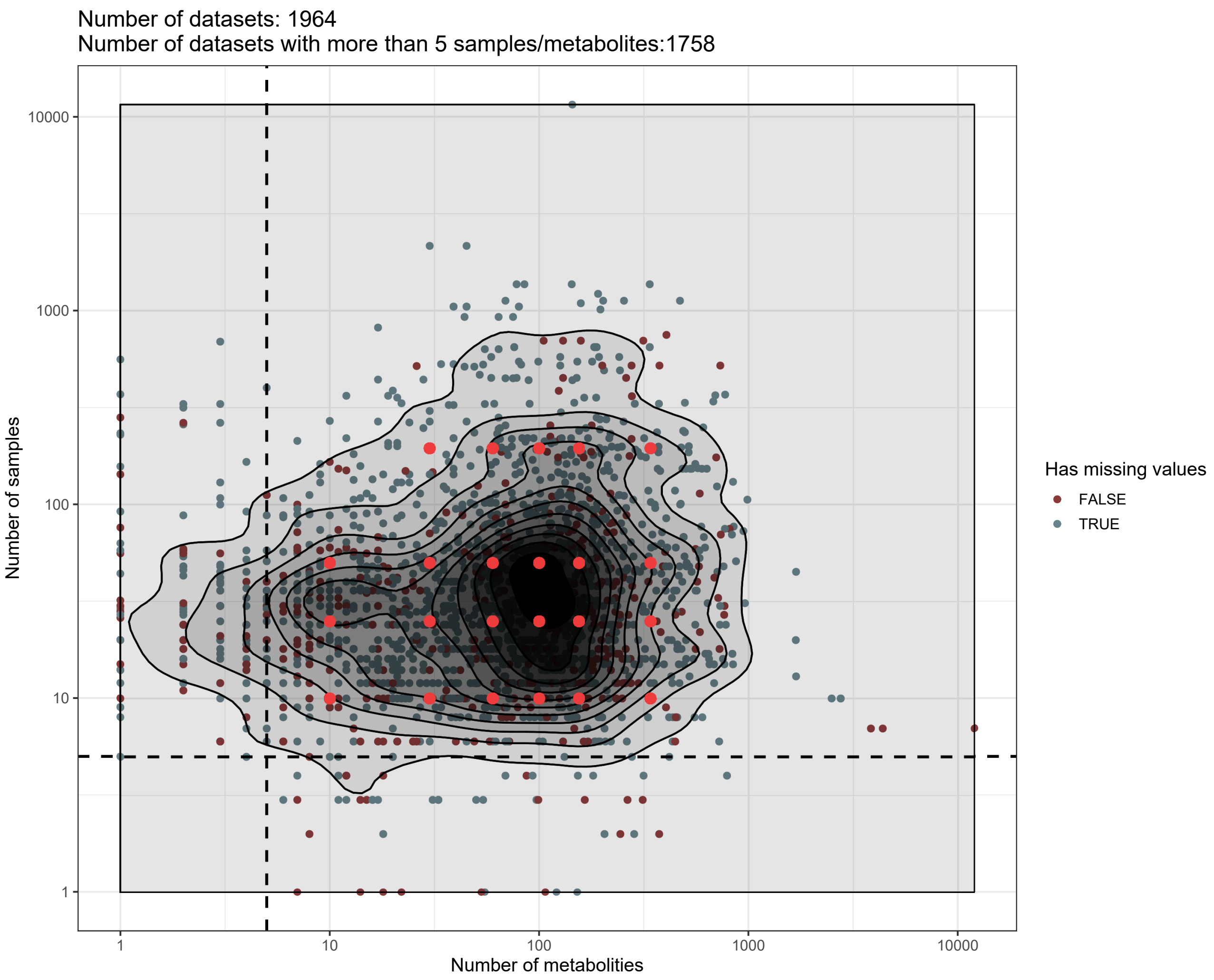

Figure S4: **Selected MW datasets within a computed grid (red points). Brown and oceanblue points represent datasets without and with missing values (respectively). The contours and shades represent the 2D kernel estimator of density. The dashed line represents the threshold of 5 samples and metabolites, respectively. Both X- and Y-axis are presented in the log-scale.**

```
  capture.output()

table_string[4] <- "\\centering \\caption{\\textbf{Datasets selected for amputation and MVIA benchmark.}}"
table_string[5:7] <-  "\\begin{tabular}{cc|ccc|ccc|c} \\hline & & & Samples & & & Compounds & & \\\\ & Stu
table_string[6:7] <- NA
table_string[34] <- "\\label{tab:grid_datasets} \\end{table}"
table_string[1:2] <- NA

table_string <- na.omit(table_string)
cat(table_string)
```

SI3.2 Amputation

Next, we amputed each grid point 200 times based on a given missing value scheme. Each iteration produced a modified dataset with different missing values. After generating the amputed datasets, the next step was to filter and reject any datasets that had more than 40% missing values in any metabolite. This ensured that only datasets with a manageable level of missing data were considered for further analysis. Once the excessive missing values were removed, we assessed if any datasets remained. If there was at least one dataset left, we then selected the case where the overall missing ratio was closest to the assumed value. This selection process aimed to find the dataset that best represented the expected level of missingness for subsequent analysis or modeling.

Below, we provide detailed descriptions of the amputation schemes for MCAR, MAR, and MNAR. In each scheme, the dataset is amputed to ensure that the total number of missing values falls within a 40% threshold, and the missing values are distributed randomly across the columns.

Table S2: **Datasets selected for amputation and MVIA benchmark.**

		Samples			Compounds			
	Study Name	actual	grid	difference	actual	grid	difference	Total Difference
1	AN003904	9	10	-1	10	10	0	-1
2	AN002735	6	10	-4	26	35	-9	-13
3	AN000391	6	10	-4	58	65	-7	-11
4	AN003607	9	10	-1	91	105	-14	-15
5	AN002655	7	10	-3	147	160	-13	-16
6	AN002186	10	10	0	249	370	-121	-121
7	AN000247	20	25	-5	9	10	-1	-6
8	AN003642	24	25	-1	31	35	-4	-5
9	AN000854	20	25	-5	57	65	-8	-13
10	AN000001	24	25	-1	102	105	-3	-4
11	AN003627	12	25	-13	157	160	-3	-16
12	AN003270	25	25	0	364	370	-6	-6
13	AN001109	45	50	-5	8	10	-2	-7
14	AN000764	48	50	-2	30	35	-5	-7
15	AN000763	48	50	-2	62	65	-3	-5
16	AN000575	42	50	-8	92	105	-13	-21
17	AN002771	30	50	-20	157	160	-3	-23
18	AN003270	25	50	-25	364	370	-6	-31
19	AN000874	147	195	-48	31	35	-4	-52
20	AN000874	147	195	-48	31	65	-34	-82
21	AN002512	187	195	-8	66	105	-39	-47
22	AN000620	192	195	-3	152	160	-8	-11
23	AN002984	178	195	-17	270	370	-100	-117

3.2.1 MCAR

MCAR mechanism is implemented within *ampute_MCAR* function as follows:

1. Generate a random vector from a multinomial distribution to determine the number of missing values per column.
2. Iteratively distribute the excess number of missing values above the threshold among the columns.
3. Randomly ampute values according to the assigned numbers of missing values per column.

3.2.2 MAR

Our implementation within *ampute_MAR* function follows the steps:

1. Generate a random vector from a multinomial distribution to determine the number of missing values per column, excluding one randomly selected complete column.
2. Calculate the excess of missing values above the threshold.
3. Enter a loop that continues as long as there is an excess of missing values.

- Randomly select columns with missing values below the threshold and increment their missing values by the total sum of excess values.
- Set the missing values in columns exceeding the threshold to the threshold value.

4. Once the number of missing values for all columns is determined, we proceed to perform data amputation and for each column separately we execute the following steps:

- Select a random number k of columns without missing values to sample from.
- Let $M = (M_1, M_2, \ldots, M_k)$ be the sampled columns. We calculate vector of scaled sums S as follows

$$S = M\alpha$$

where $\alpha = (\alpha_1, \ldots, \alpha_k)$ is a random vector of scales such that $\alpha_i \sim N(0, 1)$ for $i \in 1, \ldots, k$.

- Assign missing values to the corresponding rows in the column, based on the order of the scaled sum.

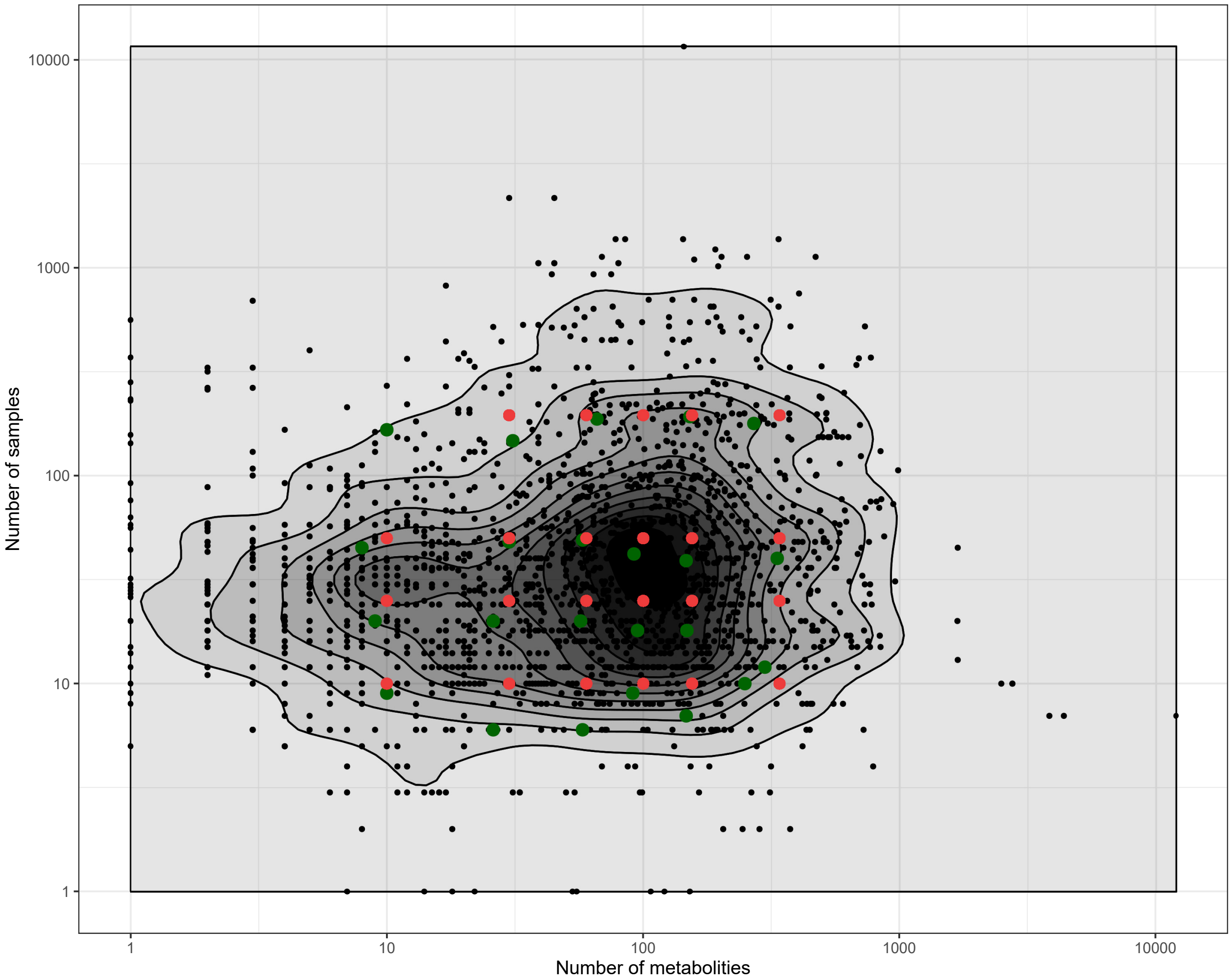

Figure S5: **Selected datasets (green) with a computed grid (red) in log-scale.**

3.2.3 MNAR

Metabolomics version of MNAR mechanism is related to LOD (limit of detection, where the missing values are measurements of intensity below the sensitivity of the measuring device). The function *ampute_MNAR* follows the following steps:

1. Generate a random vector from a multinomial distribution to determine the number of missing values per column.
2. Iteratively distribute the excess number of missing values above the threshold among the columns.
3. Remove the observations with the lowest values in each column. This ensures that the missing values are introduced in a systematic manner based on the order of values within each column.

3.2.4 Mixtures

In cases where multiple mechanisms are present, the data is amputed independently for different missing value patterns via the function *simulate_miss_value*. It is possible that more than one pattern attempts to remove the same observation, resulting in an overall missing ratio lower than the assumed value.

SI3.3 Imputation

In order to impute missing values in datasets, we utilized the implementations of the methods listed in Table S5. Each technique was invoked following the *safe imputation* strategy. This strategy involves attempting to impute the missing values using the provided imputing function running it with the default parameters. The process is repeated a maximum of 3 times, or until successful imputation is achieved. If successful imputation is achieved, the function returns the imputed dataset. However, if imputation fails in all attempts, the function returns the original dataset without any modifications. By employing *safe imputation* strategy, the function aims to offer a reliable imputation solution while taking into account potential challenges or errors that may arise during the imputation process. Moreover, each method has 1 minute time limit for evaluation. Thus, we identify three different types of imputation error:

- computional - method failed to impute data in each iteration of safe imputation, i.e. the data still contains missing values,
- modification - considerable modification of the original observations that were not missing (difference above $2.220446e - 15$),
- timeout - method failed to impute data in two minutes.

We have not scaled the data before the imputation.

SI4 Performance measure: NRMSE

In our study, we employed normalized root mean squared error (NRMSE) as the chosen performance measure.

Let $x = (x_1, \ldots, x_n)$ denote a vector of all observed values that have been removed from the data where n is a total number of missing observations. Let $\hat{x} = (\hat{x}_1, \ldots, \hat{x}_n)$ be a vector of imputed values corresponding to x.

$$NRMSE = \sqrt{\frac{1}{n}\sum_{i=1}^{n}\frac{(x_i - \hat{x}_i)^2}{\mathrm{var}(x)}}$$

We have chosen NRMSE, because it allows comparison of MVIAs regardless of the scale in the original data.

SI5 Results of simulation

Table S3: **Imputation results performed on simulated data**. The *MCAR, MAR, MNAR, total MV* columns indicate the fraction of missing values of a given type in the simulated data. The *NRMSE* column represents the Normalized Root Mean Square Error, which quantifies the accuracy of the imputation method compared to the true values. If the algorithm failed to converge, NRMSE has NA value. The *computation time* column denotes the time taken by the imputation algorithm to process the data. Finally, the *fraction of successful computation* column displays the proportion of cases where the imputation process was completed successfully without errors.

method	MCAR	MAR	MNAR	% of missing values	median NRMSE	computation time [s]	fraction of successful computation
amelia	0.00	0.00	0.05	0.0500	NA	12.9925	0.1739
amelia	0.00	0.00	0.10	0.1000	NA	13.3757	0.1304
amelia	0.00	0.05	0.00	0.0500	NA	13.1977	0.1739
amelia	0.00	0.05	0.05	0.0990	NA	13.5215	0.1304
amelia	0.00	0.10	0.00	0.1000	NA	13.1789	0.1304
amelia	0.05	0.00	0.00	0.0500	NA	12.9588	0.2174
amelia	0.05	0.00	0.05	0.0992	NA	13.5396	0.1739
amelia	0.05	0.05	0.00	0.0994	NA	13.2528	0.1739
amelia	0.05	0.05	0.05	0.1453	NA	13.5803	0.1304
amelia	0.10	0.00	0.00	0.1000	NA	13.1501	0.2174
areg	0.00	0.00	0.05	0.0500	NA	1.0099	0.9565
areg	0.00	0.00	0.10	0.1000	NA	2.0268	0.9565
areg	0.00	0.05	0.00	0.0500	NA	1.0119	0.9565
areg	0.00	0.05	0.05	0.0990	NA	1.4160	0.9565
areg	0.00	0.10	0.00	0.1000	0.7959	1.4810	1.0000
areg	0.05	0.00	0.00	0.0500	NA	1.0728	0.9565
areg	0.05	0.00	0.05	0.0992	0.8127	1.6789	1.0000
areg	0.05	0.05	0.00	0.0994	0.7038	1.5927	1.0000
areg	0.05	0.05	0.05	0.1453	0.8587	1.8542	1.0000
areg	0.10	0.00	0.00	0.1000	0.7439	2.0304	1.0000
bayesmetab	0.00	0.00	0.05	0.0500	NA	0.5563	0.1739
bayesmetab	0.00	0.00	0.10	0.1000	NA	0.5662	0.2174
bayesmetab	0.00	0.05	0.00	0.0500	NA	0.5582	0.2174
bayesmetab	0.00	0.05	0.05	0.0990	NA	0.5709	0.2174
bayesmetab	0.00	0.10	0.00	0.1000	NA	0.5648	0.2174
bayesmetab	0.05	0.00	0.00	0.0500	NA	0.5611	0.2174
bayesmetab	0.05	0.00	0.05	0.0992	NA	0.5694	0.2174
bayesmetab	0.05	0.05	0.00	0.0994	NA	0.5697	0.2174
bayesmetab	0.05	0.05	0.05	0.1453	NA	0.5743	0.2174
bayesmetab	0.10	0.00	0.00	0.1000	NA	0.5625	0.1739
bcv_svd	0.00	0.00	0.05	0.0500	2.6871	0.0090	1.0000
bcv_svd	0.00	0.00	0.10	0.1000	2.4745	0.0095	1.0000
bcv_svd	0.00	0.05	0.00	0.0500	0.6927	0.0097	1.0000
bcv_svd	0.00	0.05	0.05	0.0990	0.7781	0.0097	1.0000
bcv_svd	0.00	0.10	0.00	0.1000	0.7828	0.0095	1.0000
bcv_svd	0.05	0.00	0.00	0.0500	0.5814	0.0095	1.0000
bcv_svd	0.05	0.00	0.05	0.0992	0.8754	0.0095	1.0000
bcv_svd	0.05	0.05	0.00	0.0994	0.6158	0.0090	1.0000
bcv_svd	0.05	0.05	0.05	0.1453	0.7829	0.0091	1.0000
bcv_svd	0.10	0.00	0.00	0.1000	0.6059	0.0097	1.0000
bpca	0.00	0.00	0.05	0.0500	1.2423	0.0572	1.0000
bpca	0.00	0.00	0.10	0.1000	1.7020	0.0831	1.0000
bpca	0.00	0.05	0.00	0.0500	0.3845	0.0532	1.0000
bpca	0.00	0.05	0.05	0.0990	0.5359	0.0852	1.0000
bpca	0.00	0.10	0.00	0.1000	0.3625	0.0700	1.0000
bpca	0.05	0.00	0.00	0.0500	0.3865	0.0632	1.0000

Table S3: **Imputation results performed on simulated data**. The *MCAR, MAR, MNAR, total MV* columns indicate the fraction of missing values of a given type in the simulated data. The *NRMSE* column represents the Normalized Root Mean Square Error, which quantifies the accuracy of the imputation method compared to the true values. If the algorithm failed to converge, NRMSE has NA value. The *computation time* column denotes the time taken by the imputation algorithm to process the data. Finally, the *fraction of successful computation* column displays the proportion of cases where the imputation process was completed successfully without errors. *(continued)*

method	MCAR	MAR	MNAR	% of missing values	median NRMSE	computation time [s]	fraction of successful computation
bpca	0.05	0.00	0.05	0.0992	0.5102	0.0656	1.0000
bpca	0.05	0.05	0.00	0.0994	0.3443	0.1190	1.0000
bpca	0.05	0.05	0.05	0.1453	0.5493	0.1439	1.0000
bpca	0.10	0.00	0.00	0.1000	0.3858	0.0764	1.0000
cm	0.00	0.00	0.05	0.0500	0.4921	1.4858	1.0000
cm	0.00	0.00	0.10	0.1000	0.5413	0.0078	1.0000
cm	0.00	0.05	0.00	0.0500	0.3488	1.4535	1.0000
cm	0.00	0.05	0.05	0.0990	0.8159	0.0140	1.0000
cm	0.00	0.10	0.00	0.1000	0.8823	0.4316	1.0000
cm	0.05	0.00	0.00	0.0500	0.4680	0.7967	1.0000
cm	0.05	0.00	0.05	0.0992	0.6371	0.8371	1.0000
cm	0.05	0.05	0.00	0.0994	0.9136	0.0084	1.0000
cm	0.05	0.05	0.05	0.1453	0.7495	0.0094	1.0000
cm	0.10	0.00	0.00	0.1000	0.8515	0.4560	1.0000
corknn	0.00	0.00	0.05	0.0500	1.0403	0.0073	1.0000
corknn	0.00	0.00	0.10	0.1000	NA	0.0101	0.9130
corknn	0.00	0.05	0.00	0.0500	0.5609	0.0072	1.0000
corknn	0.00	0.05	0.05	0.0990	NA	0.0110	0.9565
corknn	0.00	0.10	0.00	0.1000	0.5039	0.0109	1.0000
corknn	0.05	0.00	0.00	0.0500	0.4575	0.0071	1.0000
corknn	0.05	0.00	0.05	0.0992	0.4553	0.0128	1.0000
corknn	0.05	0.05	0.00	0.0994	0.5497	0.0105	1.0000
corknn	0.05	0.05	0.05	0.1453	NA	0.0130	0.9130
corknn	0.10	0.00	0.00	0.1000	0.5032	0.0107	1.0000
eucknn	0.00	0.00	0.05	0.0500	1.5321	0.0049	1.0000
eucknn	0.00	0.00	0.10	0.1000	1.9600	0.0075	1.0000
eucknn	0.00	0.05	0.00	0.0500	0.3786	0.0052	1.0000
eucknn	0.00	0.05	0.05	0.0990	0.4763	0.0073	1.0000
eucknn	0.00	0.10	0.00	0.1000	0.5299	0.0071	1.0000
eucknn	0.05	0.00	0.00	0.0500	0.3146	0.0049	1.0000
eucknn	0.05	0.00	0.05	0.0992	0.4739	0.0075	1.0000
eucknn	0.05	0.05	0.00	0.0994	0.3457	0.0072	1.0000
eucknn	0.05	0.05	0.05	0.1453	0.4580	0.0099	1.0000
eucknn	0.10	0.00	0.00	0.1000	0.3576	0.0072	1.0000
gsimp	0.00	0.00	0.05	0.0500	NA	120.0407	0.3478
gsimp	0.00	0.00	0.10	0.1000	NA	120.0677	0.3478
gsimp	0.00	0.05	0.00	0.0500	NA	120.0291	0.4348
gsimp	0.00	0.05	0.05	0.0990	NA	120.0550	0.3913
gsimp	0.00	0.10	0.00	0.1000	NA	120.0585	0.3478
gsimp	0.05	0.00	0.00	0.0500	NA	120.0494	0.4348
gsimp	0.05	0.00	0.05	0.0992	NA	120.0461	0.3913
gsimp	0.05	0.05	0.00	0.0994	NA	120.0384	0.3913
gsimp	0.05	0.05	0.05	0.1453	NA	120.0559	0.3913
gsimp	0.10	0.00	0.00	0.1000	NA	120.0599	0.3913
halfmin	0.00	0.00	0.05	0.0500	0.4910	0.0045	1.0000
halfmin	0.00	0.00	0.10	0.1000	0.4919	0.0050	1.0000
halfmin	0.00	0.05	0.00	0.0500	0.9978	0.0051	1.0000
halfmin	0.00	0.05	0.05	0.0990	0.9108	0.0053	1.0000
halfmin	0.00	0.10	0.00	0.1000	0.9819	0.0053	1.0000

Table S3: **Imputation results performed on simulated data**. The *MCAR, MAR, MNAR, total MV* columns indicate the fraction of missing values of a given type in the simulated data. The *NRMSE* column represents the Normalized Root Mean Square Error, which quantifies the accuracy of the imputation method compared to the true values. If the algorithm failed to converge, NRMSE has NA value. The *computation time* column denotes the time taken by the imputation algorithm to process the data. Finally, the *fraction of successful computation* column displays the proportion of cases where the imputation process was completed successfully without errors. *(continued)*

**method**	**MCAR**	**MAR**	**MNAR**	**% of missing values**	**median NRMSE**	**computation time [s]**	**fraction of successful computation**
halfmin	0.05	0.00	0.00	0.0500	0.9907	0.0047	1.0000
halfmin	0.05	0.00	0.05	0.0992	0.8754	0.0057	1.0000
halfmin	0.05	0.05	0.00	0.0994	1.0065	0.0054	1.0000
halfmin	0.05	0.05	0.05	0.1453	0.9677	0.0050	1.0000
halfmin	0.10	0.00	0.00	0.1000	0.9745	0.0051	1.0000
imputation__knn	0.00	0.00	0.05	0.0500	1.8152	0.0058	1.0000
imputation__knn	0.00	0.00	0.10	0.1000	2.2725	0.0091	1.0000
imputation__knn	0.00	0.05	0.00	0.0500	0.9426	0.0059	1.0000
imputation__knn	0.00	0.05	0.05	0.0990	0.9075	0.0089	1.0000
imputation__knn	0.00	0.10	0.00	0.1000	0.9840	0.0091	1.0000
imputation__knn	0.05	0.00	0.00	0.0500	0.8468	0.0061	1.0000
imputation__knn	0.05	0.00	0.05	0.0992	0.8487	0.0094	1.0000
imputation__knn	0.05	0.05	0.00	0.0994	0.8870	0.0095	1.0000
imputation__knn	0.05	0.05	0.05	0.1453	0.9263	0.0122	1.0000
imputation__knn	0.10	0.00	0.00	0.1000	0.8277	0.0095	1.0000
knn	0.00	0.00	0.05	0.0500	0.6408	0.0028	1.0000
knn	0.00	0.00	0.10	0.1000	NA	0.0028	0.9565
knn	0.00	0.05	0.00	0.0500	0.8025	0.0028	1.0000
knn	0.00	0.05	0.05	0.0990	NA	0.0031	0.9565
knn	0.00	0.10	0.00	0.1000	0.7958	0.0028	1.0000
knn	0.05	0.00	0.00	0.0500	0.8136	0.0027	1.0000
knn	0.05	0.00	0.05	0.0992	0.8068	0.0028	1.0000
knn	0.05	0.05	0.00	0.0994	0.7973	0.0029	1.0000
knn	0.05	0.05	0.05	0.1453	0.8251	0.0031	1.0000
knn	0.10	0.00	0.00	0.1000	0.7854	0.0027	1.0000
mai	0.00	0.00	0.05	0.0500	NA	0.5277	0.0000
mai	0.00	0.00	0.10	0.1000	NA	4.0622	0.0870
mai	0.00	0.05	0.00	0.0500	NA	0.5282	0.0000
mai	0.00	0.05	0.05	0.0990	NA	2.4012	0.0435
mai	0.00	0.10	0.00	0.1000	NA	5.1487	0.0870
mai	0.05	0.00	0.00	0.0500	NA	0.5299	0.0000
mai	0.05	0.00	0.05	0.0992	NA	5.1256	0.0435
mai	0.05	0.05	0.00	0.0994	NA	4.0084	0.0435
mai	0.05	0.05	0.05	0.1453	NA	6.2983	0.0435
mai	0.10	0.00	0.00	0.1000	NA	7.0693	0.0870
mean	0.00	0.00	0.05	0.0500	2.6871	0.0054	1.0000
mean	0.00	0.00	0.10	0.1000	2.4745	0.0054	1.0000
mean	0.00	0.05	0.00	0.0500	0.6927	0.0052	1.0000
mean	0.00	0.05	0.05	0.0990	0.7781	0.0061	1.0000
mean	0.00	0.10	0.00	0.1000	0.7828	0.0052	1.0000
mean	0.05	0.00	0.00	0.0500	0.5814	0.0065	1.0000
mean	0.05	0.00	0.05	0.0992	0.8754	0.0060	1.0000
mean	0.05	0.05	0.00	0.0994	0.6158	0.0056	1.0000
mean	0.05	0.05	0.05	0.1453	0.7829	0.0055	1.0000
mean	0.10	0.00	0.00	0.1000	0.6059	0.0057	1.0000
median	0.00	0.00	0.05	0.0500	2.5460	0.0053	1.0000
median	0.00	0.00	0.10	0.1000	2.3068	0.0048	1.0000
median	0.00	0.05	0.00	0.0500	0.7085	0.0047	1.0000

Table S3: **Imputation results performed on simulated data**. The *MCAR, MAR, MNAR, total MV* columns indicate the fraction of missing values of a given type in the simulated data. The *NRMSE* column represents the Normalized Root Mean Square Error, which quantifies the accuracy of the imputation method compared to the true values. If the algorithm failed to converge, NRMSE has NA value. The *computation time* column denotes the time taken by the imputation algorithm to process the data. Finally, the *fraction of successful computation* column displays the proportion of cases where the imputation process was completed successfully without errors. *(continued)*

method	MCAR	MAR	MNAR	% of missing values	median NRMSE	computation time [s]	fraction of successful computation
median	0.00	0.05	0.05	0.0990	0.7464	0.0047	1.0000
median	0.00	0.10	0.00	0.1000	0.7359	0.0047	1.0000
median	0.05	0.00	0.00	0.0500	0.5545	0.0053	1.0000
median	0.05	0.00	0.05	0.0992	0.8475	0.0050	1.0000
median	0.05	0.05	0.00	0.0994	0.6210	0.0050	1.0000
median	0.05	0.05	0.05	0.1453	0.7967	0.0048	1.0000
median	0.10	0.00	0.00	0.1000	0.6146	0.0052	1.0000
metabimpute_bpca	0.00	0.00	0.05	0.0500	2.6871	0.0902	1.0000
metabimpute_bpca	0.00	0.00	0.10	0.1000	2.0300	0.1419	1.0000
metabimpute_bpca	0.00	0.05	0.00	0.0500	NA	0.1309	0.9565
metabimpute_bpca	0.00	0.05	0.05	0.0990	0.4894	0.1588	1.0000
metabimpute_bpca	0.00	0.10	0.00	0.1000	0.4579	0.1607	1.0000
metabimpute_bpca	0.05	0.00	0.00	0.0500	NA	0.1345	0.9565
metabimpute_bpca	0.05	0.00	0.05	0.0992	0.4684	0.1404	1.0000
metabimpute_bpca	0.05	0.05	0.00	0.0994	0.3051	0.1539	1.0000
metabimpute_bpca	0.05	0.05	0.05	0.1453	0.5455	0.1576	1.0000
metabimpute_bpca	0.10	0.00	0.00	0.1000	0.4098	0.1585	1.0000
metabimpute_gsimp	0.00	0.00	0.05	0.0500	NA	24.2895	0.8696
metabimpute_gsimp	0.00	0.00	0.10	0.1000	NA	24.8597	0.8261
metabimpute_gsimp	0.00	0.05	0.00	0.0500	NA	22.4674	0.8696
metabimpute_gsimp	0.00	0.05	0.05	0.0990	NA	25.5058	0.9130
metabimpute_gsimp	0.00	0.10	0.00	0.1000	NA	25.2436	0.9130
metabimpute_gsimp	0.05	0.00	0.00	0.0500	NA	24.3348	0.8696
metabimpute_gsimp	0.05	0.00	0.05	0.0992	NA	24.3677	0.8696
metabimpute_gsimp	0.05	0.05	0.00	0.0994	NA	25.1114	0.9130
metabimpute_gsimp	0.05	0.05	0.05	0.1453	NA	23.7031	0.9565
metabimpute_gsimp	0.10	0.00	0.00	0.1000	NA	24.3488	0.9130
metabimpute_halfmin	0.00	0.00	0.05	0.0500	0.4910	0.0047	1.0000
metabimpute_halfmin	0.00	0.00	0.10	0.1000	0.4919	0.0047	1.0000
metabimpute_halfmin	0.00	0.05	0.00	0.0500	0.9978	0.0045	1.0000
metabimpute_halfmin	0.00	0.05	0.05	0.0990	0.9108	0.0046	1.0000
metabimpute_halfmin	0.00	0.10	0.00	0.1000	0.9819	0.0046	1.0000
metabimpute_halfmin	0.05	0.00	0.00	0.0500	0.9907	0.0046	1.0000
metabimpute_halfmin	0.05	0.00	0.05	0.0992	0.8754	0.0049	1.0000
metabimpute_halfmin	0.05	0.05	0.00	0.0994	1.0065	0.0047	1.0000
metabimpute_halfmin	0.05	0.05	0.05	0.1453	0.9677	0.0045	1.0000
metabimpute_halfmin	0.10	0.00	0.00	0.1000	0.9745	0.0049	1.0000
metabimpute_mean	0.00	0.00	0.05	0.0500	2.6871	0.0042	1.0000
metabimpute_mean	0.00	0.00	0.10	0.1000	2.4745	0.0043	1.0000
metabimpute_mean	0.00	0.05	0.00	0.0500	0.6927	0.0042	1.0000
metabimpute_mean	0.00	0.05	0.05	0.0990	0.7781	0.0042	1.0000
metabimpute_mean	0.00	0.10	0.00	0.1000	0.7828	0.0042	1.0000
metabimpute_mean	0.05	0.00	0.00	0.0500	0.5814	0.0044	1.0000
metabimpute_mean	0.05	0.00	0.05	0.0992	0.8754	0.0047	1.0000
metabimpute_mean	0.05	0.05	0.00	0.0994	0.6158	0.0043	1.0000
metabimpute_mean	0.05	0.05	0.05	0.1453	0.7829	0.0044	1.0000
metabimpute_mean	0.10	0.00	0.00	0.1000	0.6059	0.0044	1.0000
metabimpute_median	0.00	0.00	0.05	0.0500	2.5460	0.0054	1.0000

Table S3: **Imputation results performed on simulated data**. The *MCAR, MAR, MNAR, total MV* columns indicate the fraction of missing values of a given type in the simulated data. The *NRMSE* column represents the Normalized Root Mean Square Error, which quantifies the accuracy of the imputation method compared to the true values. If the algorithm failed to converge, NRMSE has NA value. The *computation time* column denotes the time taken by the imputation algorithm to process the data. Finally, the *fraction of successful computation* column displays the proportion of cases where the imputation process was completed successfully without errors. *(continued)*

method	MCAR	MAR	MNAR	% of missing values	median NRMSE	computation time [s]	fraction of successful computation
metabimpute_median	0.00	0.00	0.10	0.1000	2.3068	0.0055	1.0000
metabimpute_median	0.00	0.05	0.00	0.0500	0.7085	0.0055	1.0000
metabimpute_median	0.00	0.05	0.05	0.0990	0.7464	0.0056	1.0000
metabimpute_median	0.00	0.10	0.00	0.1000	0.7359	0.0057	1.0000
metabimpute_median	0.05	0.00	0.00	0.0500	0.5545	0.0053	1.0000
metabimpute_median	0.05	0.00	0.05	0.0992	0.8475	0.0054	1.0000
metabimpute_median	0.05	0.05	0.00	0.0994	0.6210	0.0055	1.0000
metabimpute_median	0.05	0.05	0.05	0.1453	0.7967	0.0053	1.0000
metabimpute_median	0.10	0.00	0.00	0.1000	0.6146	0.0056	1.0000
metabimpute_min	0.00	0.00	0.05	0.0500	0.4339	0.0041	1.0000
metabimpute_min	0.00	0.00	0.10	0.1000	0.4259	0.0041	1.0000
metabimpute_min	0.00	0.05	0.00	0.0500	0.9408	0.0041	1.0000
metabimpute_min	0.00	0.05	0.05	0.0990	0.8196	0.0041	1.0000
metabimpute_min	0.00	0.10	0.00	0.1000	0.9279	0.0040	1.0000
metabimpute_min	0.05	0.00	0.00	0.0500	0.8964	0.0039	1.0000
metabimpute_min	0.05	0.00	0.05	0.0992	0.7846	0.0040	1.0000
metabimpute_min	0.05	0.05	0.00	0.0994	0.9136	0.0041	1.0000
metabimpute_min	0.05	0.05	0.05	0.1453	0.8982	0.0041	1.0000
metabimpute_min	0.10	0.00	0.00	0.1000	0.9047	0.0041	1.0000
metabimpute_qrilc	0.00	0.00	0.05	0.0500	0.7126	0.0454	1.0000
metabimpute_qrilc	0.00	0.00	0.10	0.1000	0.7013	0.0457	1.0000
metabimpute_qrilc	0.00	0.05	0.00	0.0500	1.0020	0.0450	1.0000
metabimpute_qrilc	0.00	0.05	0.05	0.0990	0.9373	0.0453	1.0000
metabimpute_qrilc	0.00	0.10	0.00	0.1000	1.0035	0.0462	1.0000
metabimpute_qrilc	0.05	0.00	0.00	0.0500	0.9656	0.0453	1.0000
metabimpute_qrilc	0.05	0.00	0.05	0.0992	0.9883	0.0451	1.0000
metabimpute_qrilc	0.05	0.05	0.00	0.0994	1.0086	0.0456	1.0000
metabimpute_qrilc	0.05	0.05	0.05	0.1453	0.9766	0.0464	1.0000
metabimpute_qrilc	0.10	0.00	0.00	0.1000	0.9954	0.0453	1.0000
metabimpute_rf	0.00	0.00	0.05	0.0500	NA	0.5623	0.9565
metabimpute_rf	0.00	0.00	0.10	0.1000	1.6193	1.0782	1.0000
metabimpute_rf	0.00	0.05	0.00	0.0500	0.4081	0.6074	1.0000
metabimpute_rf	0.00	0.05	0.05	0.0990	0.4296	0.8868	1.0000
metabimpute_rf	0.00	0.10	0.00	0.1000	NA	0.8703	0.9565
metabimpute_rf	0.05	0.00	0.00	0.0500	0.3076	0.8687	1.0000
metabimpute_rf	0.05	0.00	0.05	0.0992	0.4852	0.6674	1.0000
metabimpute_rf	0.05	0.05	0.00	0.0994	NA	0.6697	0.9565
metabimpute_rf	0.05	0.05	0.05	0.1453	NA	0.6689	0.9565
metabimpute_rf	0.10	0.00	0.00	0.1000	0.3071	0.6675	1.0000
metabimpute_zero	0.00	0.00	0.05	0.0500	1.1119	0.0050	1.0000
metabimpute_zero	0.00	0.00	0.10	0.1000	1.0930	0.0051	1.0000
metabimpute_zero	0.00	0.05	0.00	0.0500	1.0951	0.0049	1.0000
metabimpute_zero	0.00	0.05	0.05	0.0990	1.0570	0.0048	1.0000
metabimpute_zero	0.00	0.10	0.00	0.1000	1.0710	0.0047	1.0000
metabimpute_zero	0.05	0.00	0.00	0.0500	1.0795	0.0052	1.0000
metabimpute_zero	0.05	0.00	0.05	0.0992	1.0580	0.0050	1.0000
metabimpute_zero	0.05	0.05	0.00	0.0994	1.0608	0.0052	1.0000
metabimpute_zero	0.05	0.05	0.05	0.1453	1.0634	0.0052	1.0000
metabimpute_zero	0.10	0.00	0.00	0.1000	1.0672	0.0050	1.0000

Table S3: **Imputation results performed on simulated data**. The *MCAR, MAR, MNAR, total MV* columns indicate the fraction of missing values of a given type in the simulated data. The *NRMSE* column represents the Normalized Root Mean Square Error, which quantifies the accuracy of the imputation method compared to the true values. If the algorithm failed to converge, NRMSE has NA value. The *computation time* column denotes the time taken by the imputation algorithm to process the data. Finally, the *fraction of successful computation* column displays the proportion of cases where the imputation process was completed successfully without errors. *(continued)*

method	MCAR	MAR	MNAR	% of missing values	median NRMSE	computation time [s]	fraction of successful computation
mice_cart	0.00	0.00	0.05	0.0500	NA	21.7016	0.4783
mice_cart	0.00	0.00	0.10	0.1000	NA	25.7124	0.4348
mice_cart	0.00	0.05	0.00	0.0500	NA	21.7074	0.4783
mice_cart	0.00	0.05	0.05	0.0990	NA	24.9421	0.3913
mice_cart	0.00	0.10	0.00	0.1000	NA	24.6883	0.4348
mice_cart	0.05	0.00	0.00	0.0500	NA	23.6996	0.4348
mice_cart	0.05	0.00	0.05	0.0992	NA	25.0703	0.3913
mice_cart	0.05	0.05	0.00	0.0994	NA	25.2453	0.4348
mice_cart	0.05	0.05	0.05	0.1453	NA	25.0534	0.3913
mice_cart	0.10	0.00	0.00	0.1000	NA	22.7914	0.4348
mice_pmm	0.00	0.00	0.05	0.0500	NA	1.1990	0.3913
mice_pmm	0.00	0.00	0.10	0.1000	NA	1.4025	0.3913
mice_pmm	0.00	0.05	0.00	0.0500	NA	1.2298	0.3913
mice_pmm	0.00	0.05	0.05	0.0990	NA	1.4040	0.3478
mice_pmm	0.00	0.10	0.00	0.1000	NA	1.3438	0.3913
mice_pmm	0.05	0.00	0.00	0.0500	NA	1.1705	0.3913
mice_pmm	0.05	0.00	0.05	0.0992	NA	0.6333	0.3478
mice_pmm	0.05	0.05	0.00	0.0994	NA	1.3543	0.3913
mice_pmm	0.05	0.05	0.05	0.1453	NA	0.6879	0.3478
mice_pmm	0.10	0.00	0.00	0.1000	NA	1.3813	0.3913
mice_rf	0.00	0.00	0.05	0.0500	NA	20.6102	0.4783
mice_rf	0.00	0.00	0.10	0.1000	NA	24.9753	0.4348
mice_rf	0.00	0.05	0.00	0.0500	NA	20.2963	0.4783
mice_rf	0.00	0.05	0.05	0.0990	NA	24.7950	0.3913
mice_rf	0.00	0.10	0.00	0.1000	NA	22.9709	0.4348
mice_rf	0.05	0.00	0.00	0.0500	NA	21.5951	0.4348
mice_rf	0.05	0.00	0.05	0.0992	NA	24.0341	0.3913
mice_rf	0.05	0.05	0.00	0.0994	NA	24.8171	0.4348
mice_rf	0.05	0.05	0.05	0.1453	NA	24.8761	0.3913
mice_rf	0.10	0.00	0.00	0.1000	NA	23.3092	0.4348
min	0.00	0.00	0.05	0.0500	0.4339	0.0058	1.0000
min	0.00	0.00	0.10	0.1000	0.4259	0.0062	1.0000
min	0.00	0.05	0.00	0.0500	0.9408	0.0053	1.0000
min	0.00	0.05	0.05	0.0990	0.8196	0.0056	1.0000
min	0.00	0.10	0.00	0.1000	0.9279	0.0056	1.0000
min	0.05	0.00	0.00	0.0500	0.8964	0.0057	1.0000
min	0.05	0.00	0.05	0.0992	0.7846	0.0053	1.0000
min	0.05	0.05	0.00	0.0994	0.9136	0.0057	1.0000
min	0.05	0.05	0.05	0.1453	0.8982	0.0060	1.0000
min	0.10	0.00	0.00	0.1000	0.9047	0.0056	1.0000
missforest	0.00	0.00	0.05	0.0500	NA	1.2764	0.9565
missforest	0.00	0.00	0.10	0.1000	NA	1.6842	0.9565
missforest	0.00	0.05	0.00	0.0500	NA	0.9215	0.9565
missforest	0.00	0.05	0.05	0.0990	NA	1.3695	0.9565
missforest	0.00	0.10	0.00	0.1000	NA	2.2154	0.9565
missforest	0.05	0.00	0.00	0.0500	NA	1.3955	0.9565
missforest	0.05	0.00	0.05	0.0992	NA	1.4105	0.9565
missforest	0.05	0.05	0.00	0.0994	NA	1.4583	0.9565

Table S3: **Imputation results performed on simulated data**. The *MCAR, MAR, MNAR, total MV* columns indicate the fraction of missing values of a given type in the simulated data. The *NRMSE* column represents the Normalized Root Mean Square Error, which quantifies the accuracy of the imputation method compared to the true values. If the algorithm failed to converge, NRMSE has NA value. The *computation time* column denotes the time taken by the imputation algorithm to process the data. Finally, the *fraction of successful computation* column displays the proportion of cases where the imputation process was completed successfully without errors. *(continued)*

method	MCAR	MAR	MNAR	% of missing values	median NRMSE	computation time [s]	fraction of successful computation
missforest	0.05	0.05	0.05	0.1453	NA	1.7926	0.9565
missforest	0.10	0.00	0.00	0.1000	NA	2.3435	0.9565
missmda_em	0.00	0.00	0.05	0.0500	1.1956	0.0186	1.0000
missmda_em	0.00	0.00	0.10	0.1000	2.1479	0.0318	1.0000
missmda_em	0.00	0.05	0.00	0.0500	0.3362	0.0202	1.0000
missmda_em	0.00	0.05	0.05	0.0990	0.3854	0.0359	1.0000
missmda_em	0.00	0.10	0.00	0.1000	0.3153	0.0340	1.0000
missmda_em	0.05	0.00	0.00	0.0500	0.3382	0.0164	1.0000
missmda_em	0.05	0.00	0.05	0.0992	0.3778	0.0207	1.0000
missmda_em	0.05	0.05	0.00	0.0994	0.4521	0.0334	1.0000
missmda_em	0.05	0.05	0.05	0.1453	0.3983	0.0364	1.0000
missmda_em	0.10	0.00	0.00	0.1000	0.3655	0.0196	1.0000
mnmf	0.00	0.00	0.05	0.0500	NA	2.9699	0.3478
mnmf	0.00	0.00	0.10	0.1000	NA	2.9654	0.3478
mnmf	0.00	0.05	0.00	0.0500	NA	3.4958	0.3478
mnmf	0.00	0.05	0.05	0.0990	NA	2.9192	0.3478
mnmf	0.00	0.10	0.00	0.1000	NA	2.9134	0.3478
mnmf	0.05	0.00	0.00	0.0500	NA	2.9479	0.3478
mnmf	0.05	0.00	0.05	0.0992	NA	2.9227	0.3478
mnmf	0.05	0.05	0.00	0.0994	NA	2.9654	0.3478
mnmf	0.05	0.05	0.05	0.1453	NA	2.9092	0.3478
mnmf	0.10	0.00	0.00	0.1000	NA	2.9165	0.3478
nipals	0.00	0.00	0.05	0.0500	1.8038	0.0038	1.0000
nipals	0.00	0.00	0.10	0.1000	2.3064	0.0039	1.0000
nipals	0.00	0.05	0.00	0.0500	0.5503	0.0038	1.0000
nipals	0.00	0.05	0.05	0.0990	0.7223	0.0040	1.0000
nipals	0.00	0.10	0.00	0.1000	0.6237	0.0039	1.0000
nipals	0.05	0.00	0.00	0.0500	0.4243	0.0040	1.0000
nipals	0.05	0.00	0.05	0.0992	0.7200	0.0037	1.0000
nipals	0.05	0.05	0.00	0.0994	0.5169	0.0040	1.0000
nipals	0.05	0.05	0.05	0.1453	0.6934	0.0036	1.0000
nipals	0.10	0.00	0.00	0.1000	0.5096	0.0036	1.0000
pemm	0.00	0.00	0.05	0.0500	NA	0.8330	0.7391
pemm	0.00	0.00	0.10	0.1000	NA	1.1056	0.7826
pemm	0.00	0.05	0.00	0.0500	NA	0.6006	0.8261
pemm	0.00	0.05	0.05	0.0990	NA	0.5812	0.9130
pemm	0.00	0.10	0.00	0.1000	NA	0.4064	0.8261
pemm	0.05	0.00	0.00	0.0500	NA	0.5635	0.7826
pemm	0.05	0.00	0.05	0.0992	NA	1.1127	0.8696
pemm	0.05	0.05	0.00	0.0994	NA	1.0732	0.8696
pemm	0.05	0.05	0.05	0.1453	NA	0.4744	0.9130
pemm	0.10	0.00	0.00	0.1000	NA	1.1199	0.7391
ppca	0.00	0.00	0.05	0.0500	1.3602	0.0111	1.0000
ppca	0.00	0.00	0.10	0.1000	2.2173	0.0117	1.0000
ppca	0.00	0.05	0.00	0.0500	0.3922	0.0106	1.0000
ppca	0.00	0.05	0.05	0.0990	0.5159	0.0133	1.0000
ppca	0.00	0.10	0.00	0.1000	0.4903	0.0129	1.0000
ppca	0.05	0.00	0.00	0.0500	0.3796	0.0115	1.0000

Table S3: **Imputation results performed on simulated data**. The *MCAR, MAR, MNAR, total MV* columns indicate the fraction of missing values of a given type in the simulated data. The *NRMSE* column represents the Normalized Root Mean Square Error, which quantifies the accuracy of the imputation method compared to the true values. If the algorithm failed to converge, NRMSE has NA value. The *computation time* column denotes the time taken by the imputation algorithm to process the data. Finally, the *fraction of successful computation* column displays the proportion of cases where the imputation process was completed successfully without errors. *(continued)*

method	MCAR	MAR	MNAR	% of missing values	median NRMSE	computation time [s]	fraction of successful computation
ppca	0.05	0.00	0.05	0.0992	0.4947	0.0109	1.0000
ppca	0.05	0.05	0.00	0.0994	0.3795	0.0120	1.0000
ppca	0.05	0.05	0.05	0.1453	0.6020	0.0131	1.0000
ppca	0.10	0.00	0.00	0.1000	0.3823	0.0116	1.0000
qrilc	0.00	0.00	0.05	0.0500	1.2580	0.0440	1.0000
qrilc	0.00	0.00	0.10	0.1000	1.2701	0.0442	1.0000
qrilc	0.00	0.05	0.00	0.0500	1.2488	0.0446	1.0000
qrilc	0.00	0.05	0.05	0.0990	1.0868	0.0442	1.0000
qrilc	0.00	0.10	0.00	0.1000	1.2546	0.0435	1.0000
qrilc	0.05	0.00	0.00	0.0500	1.2004	0.0439	1.0000
qrilc	0.05	0.00	0.05	0.0992	1.2070	0.0435	1.0000
qrilc	0.05	0.05	0.00	0.0994	1.2767	0.0443	1.0000
qrilc	0.05	0.05	0.05	0.1453	1.2680	0.0443	1.0000
qrilc	0.10	0.00	0.00	0.1000	1.3719	0.0441	1.0000
random	0.00	0.00	0.05	0.0500	3.0482	6.6779	1.0000
random	0.00	0.00	0.10	0.1000	3.0489	6.4951	1.0000
random	0.00	0.05	0.00	0.0500	0.9782	6.7046	1.0000
random	0.00	0.05	0.05	0.0990	0.8186	6.3674	1.0000
random	0.00	0.10	0.00	0.1000	0.9174	6.6160	1.0000
random	0.05	0.00	0.00	0.0500	0.8270	6.8615	1.0000
random	0.05	0.00	0.05	0.0992	1.0638	6.7696	1.0000
random	0.05	0.05	0.00	0.0994	0.7497	6.7144	1.0000
random	0.05	0.05	0.05	0.1453	0.8724	6.6628	1.0000
random	0.10	0.00	0.00	0.1000	0.7533	6.4986	1.0000
regimpute	0.00	0.00	0.05	0.0500	NA	14.4211	0.6957
regimpute	0.00	0.00	0.10	0.1000	NA	19.6048	0.6522
regimpute	0.00	0.05	0.00	0.0500	NA	12.9962	0.6957
regimpute	0.00	0.05	0.05	0.0990	NA	16.3930	0.6957
regimpute	0.00	0.10	0.00	0.1000	NA	17.6082	0.6957
regimpute	0.05	0.00	0.00	0.0500	NA	12.9796	0.6957
regimpute	0.05	0.00	0.05	0.0992	NA	14.0568	0.6957
regimpute	0.05	0.05	0.00	0.0994	NA	18.4814	0.6957
regimpute	0.05	0.05	0.05	0.1453	NA	15.8183	0.6522
regimpute	0.10	0.00	0.00	0.1000	NA	20.1864	0.6522
svd	0.00	0.00	0.05	0.0500	1.4892	0.0120	1.0000
svd	0.00	0.00	0.10	0.1000	2.2460	0.0171	1.0000
svd	0.00	0.05	0.00	0.0500	0.3596	0.0145	1.0000
svd	0.00	0.05	0.05	0.0990	0.5487	0.0167	1.0000
svd	0.00	0.10	0.00	0.1000	0.4847	0.0249	1.0000
svd	0.05	0.00	0.00	0.0500	0.4108	0.0121	1.0000
svd	0.05	0.00	0.05	0.0992	0.5802	0.0193	1.0000
svd	0.05	0.05	0.00	0.0994	0.3721	0.0158	1.0000
svd	0.05	0.05	0.05	0.1453	0.6966	0.0317	1.0000
svd	0.10	0.00	0.00	0.1000	0.4557	0.0197	1.0000
tknn	0.00	0.00	0.05	0.0500	1.0403	0.5476	1.0000
tknn	0.00	0.00	0.10	0.1000	NA	0.5241	0.9130
tknn	0.00	0.05	0.00	0.0500	0.5609	0.5439	1.0000
tknn	0.00	0.05	0.05	0.0990	NA	0.5232	0.9565
tknn	0.00	0.10	0.00	0.1000	0.5039	0.5263	1.0000

Table S3: **Imputation results performed on simulated data**. The *MCAR, MAR, MNAR, total MV* columns indicate the fraction of missing values of a given type in the simulated data. The *NRMSE* column represents the Normalized Root Mean Square Error, which quantifies the accuracy of the imputation method compared to the true values. If the algorithm failed to converge, NRMSE has NA value. The *computation time* column denotes the time taken by the imputation algorithm to process the data. Finally, the *fraction of successful computation* column displays the proportion of cases where the imputation process was completed successfully without errors. *(continued)*

**method**	**MCAR**	**MAR**	**MNAR**	**% of missing values**	**median NRMSE**	**computation time [s]**	**fraction of successful computation**
tknn	0.05	0.00	0.00	0.0500	0.4575	0.5313	1.0000
tknn	0.05	0.00	0.05	0.0992	0.4553	0.8046	1.0000
tknn	0.05	0.05	0.00	0.0994	0.5497	0.5342	1.0000
tknn	0.05	0.05	0.05	0.1453	NA	0.5442	0.9130
tknn	0.10	0.00	0.00	0.1000	0.5032	0.5363	1.0000
vim_knn	0.00	0.00	0.05	0.0500	1.4754	0.5605	1.0000
vim_knn	0.00	0.00	0.10	0.1000	1.5311	1.0221	1.0000
vim_knn	0.00	0.05	0.00	0.0500	0.5680	0.5438	1.0000
vim_knn	0.00	0.05	0.05	0.0990	0.4468	0.7623	1.0000
vim_knn	0.00	0.10	0.00	0.1000	0.6383	0.9962	1.0000
vim_knn	0.05	0.00	0.00	0.0500	0.4565	0.5595	1.0000
vim_knn	0.05	0.00	0.05	0.0992	0.5781	0.9111	1.0000
vim_knn	0.05	0.05	0.00	0.0994	0.4662	0.8606	1.0000
vim_knn	0.05	0.05	0.05	0.1453	0.5457	0.9910	1.0000
vim_knn	0.10	0.00	0.00	0.1000	0.4296	1.0211	1.0000
zero	0.00	0.00	0.05	0.0500	1.1119	0.0034	1.0000
zero	0.00	0.00	0.10	0.1000	1.0930	0.0035	1.0000
zero	0.00	0.05	0.00	0.0500	1.0951	0.0031	1.0000
zero	0.00	0.05	0.05	0.0990	1.0570	0.0038	1.0000
zero	0.00	0.10	0.00	0.1000	1.0710	0.0036	1.0000
zero	0.05	0.00	0.00	0.0500	1.0795	0.0033	1.0000
zero	0.05	0.00	0.05	0.0992	1.0580	0.0038	1.0000
zero	0.05	0.05	0.00	0.0994	1.0608	0.0033	1.0000
zero	0.05	0.05	0.05	0.1453	1.0634	0.0040	1.0000
zero	0.10	0.00	0.00	0.1000	1.0672	0.0038	1.0000

SI6 Summary of simulation results

To establish the best-performing MVIAs for specific missing value scenarios, we have filtered out the method with stability of 0.99 or better. Next, we excluded the results for scenarios allowing mixtures of missing values. For scenarios with only a single source of missing values, we computed median NRMSE (Supplementary Table S4 and Supplementary Figure S6).

Table S4: **Summary of simulation results**. The *MCAR, MAR, MNAR* columns contain average NRMSE of each method for appropriate type of MV.

**method**	**MAR**	**MCAR**	**MNAR**
areg	0.7959	0.7439	NA
bcv_svd	0.7377	0.5936	2.5808
bpca	0.3735	0.3861	1.4721
cm	0.6156	0.6598	0.5167
corknn	0.5324	0.4804	1.0403
eucknn	0.4542	0.3361	1.7461
halfmin	0.9898	0.9826	0.4915
imputation_knn	0.9633	0.8372	2.0438
knn	0.7991	0.7995	0.6408
mean	0.7377	0.5936	2.5808
median	0.7377	0.5936	2.5808
metabimpute_bpca	0.4579	0.4098	2.3585
metabimpute_halfmin	0.9898	0.9826	0.4915
metabimpute_mean	0.7377	0.5936	2.5808
metabimpute_median	0.7222	0.5845	2.4264
metabimpute_min	0.9343	0.9006	0.4299
metabimpute_qrilc	1.0028	0.9805	0.7070
metabimpute_rf	0.4081	0.3074	1.6193
metabimpute_zero	1.0830	1.0733	1.1024
min	0.9343	0.9006	0.4299
missmda_em	0.3258	0.3518	1.6718
nipals	0.5870	0.4669	2.0551
ppca	0.4413	0.3810	1.7888
qrilc	1.2517	1.2862	1.2640
random	0.9478	0.7901	3.0485
svd	0.4221	0.4332	1.8676
tknn	0.5324	0.4804	1.0403
vim_knn	0.6031	0.4431	1.5032
zero	1.0830	1.0733	1.1024

The outcomes from our simulation highlight that there is no singular MVIA that universally proves effective for every type of missing value MV. Instead, the selection of MVIAs should follow a meticulous analysis of the specific datasets under study. According to our findings, for the MCAR type, the most favorable results, ranked from the lowest NRMSE to higher, were observed for metabimpute_rf, eucknn, missmda_em, ppca, bpca. In the case of MAR, the optimal choices were missmda_em, bpca, svd, ppca, eucknn. Lastly, for MNAR, the top-performing methods were metabimpute_min, min, halfmin, metabimpute_halfmin, cm.

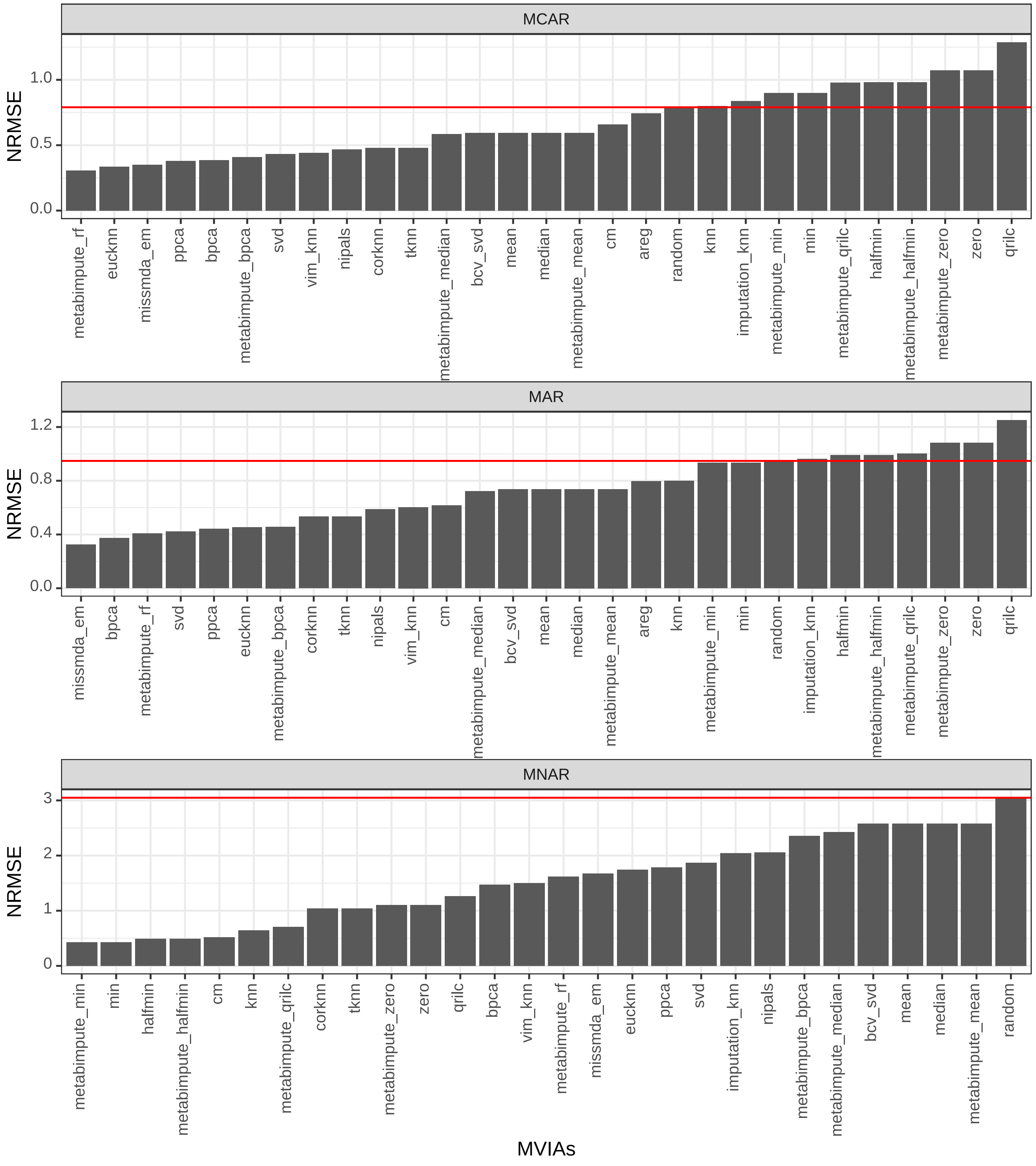

Figure S6: Normalized root mean squared error (NRMSE) of MVIAs. The red line marks the baseline MVIA: random imputation.

SI7 Example uses of imputomics

Loading *imputomics* package:

```
library(imputomics)
```

Creating data frame containing missing values:

```
idf <- data.frame(values1 = rep(c(11, 22, NA, 44, NA), 10),
                  values2 = rep(c(21, 32, 48, NA, 59), 10),
                  values3 = rep(c(37, NA, 33, 44, 32), 10))
head(idf)
```

```
##   values1 values2 values3
## 1      11      21      37
## 2      22      32      NA
## 3      NA      48      33
## 4      44      NA      44
## 5      NA      59      32
## 6      11      21      37
```

Applying various missing value imputation algorithms (MVIAs) such as mean imputation, Bayesian PCA (bpca), k-nearest neighbors (knn), and singular value decomposition (svd):

```
head(impute_mean(idf))
```

```
##    values1 values2 values3
## 1 11.00000      21    37.0
## 2 22.00000      32    36.5
## 3 25.66667      48    33.0
## 4 44.00000      40    44.0
## 5 25.66667      59    32.0
## 6 11.00000      21    37.0
```

```
head(impute_bpca(idf))
```

```
##    values1  values2  values3
## 1 11.00000 21.00000 37.00000
## 2 22.00000 32.00000 37.64877
## 3 23.04641 48.00000 33.00000
## 4 44.00000 37.95694 44.00000
## 5 29.63776 59.00000 32.00000
## 6 11.00000 21.00000 37.00000
```

```
head(impute_knn(idf))
```

```
##   values1 values2 values3
## 1    11.0    21.0      37
## 2    22.0    32.0      35
## 3    40.5    48.0      33
## 4    44.0    35.2      44
## 5    44.3    59.0      32
## 6    11.0    21.0      37
```

```
head(impute_bcv_svd(idf))
```

```
##    values1 values2 values3
## 1 11.00000      21    37.0
## 2 22.00000      32    36.5
## 3 25.66667      48    33.0
## 4 44.00000      40    44.0
## 5 25.66667      59    32.0
## 6 11.00000      21    37.0
```

For further insights into the usage of *imputomics* package, refer to the vignette.

Table S5: **Implemented MVIAs in imputomics.**

**full name**	**imputomics function**
Amelia: bootstrap EM	impute_amelia
multiple imputation additive regression	impute_areg
BayesMetab	impute_bayesmetab
Bayesian Principal Component Analysis	impute_metabimpute_bpca
Bayesian Principal Component Analysis	impute_bpca
Classification And Regression Trees	impute_mice_cart
compound minimium	impute_cm
Gibbs sampler imputation	impute_gsimp
Gibbs sampler imputation	impute_metabimpute_gsimp
half-minimum imputation	impute_halfmin
half-minimum imputation	impute_metabimpute_halfmin
k-nearest neighbors	impute_imputation_knn
k-nearest neighbors	impute_knn
k-nearest neighbors	impute_vim_knn
k-nearest neighbors correlation	impute_corknn
k-nearest neighbors euclidean	impute_eucknn
K-nearest neighbor truncation	impute_tknn
Mechanism-Aware Imputation: random_forest & Single	impute_mai
mean imputation	impute_mean
mean imputation	impute_metabimpute_mean
median imputation	impute_median
median imputation	impute_metabimpute_median
Multiple Imputation by Chained Equations Mixed	impute_mice_mixed
minimum imputation	impute_min
minimum imputation	impute_metabimpute_min
Non-negative Matrix Factorization	impute_mnmf
Non-Linear Iterative Partial Least Squares	impute_nipals
iterative PCA	impute_missmda_em
Penalized Expectation Maximization	impute_pemm
Predictive Mean Matching	impute_mice_pmm
Probabilistic Principal Component Analysis	impute_ppca
quantile regression approach for the imputation of left-censored	impute_qrilc
quantile regression approach for the imputation of left-censored	impute_metabimpute_qrilc
Random imputation	impute_random
Random Forest	impute_metabimpute_rf
Random Forest	impute_mice_rf
Random Forest	impute_missforest
glmnet ridge regression	impute_regimpute
Singular Value Decomposition	impute_bcv_svd
Singular Value Decomposition	impute_svd
zero imputation	impute_zero
zero imputation	impute_metabimpute_zero

SI8 imputomics web server

The *imputomics* application serves as an intuitive online graphical interface for the *imputomics* package. It is tailored to simplify the management of missing data using sophisticated imputation techniques, eliminating the requirement for programming skills. It is available as a web server and within R package under the function imputomics_gui().

Within the *imputomics* interface, users have the option to upload their own data or utilize pre-prepared sample data provided by us, accessible through the “Example Data” button. When uploading your data, choose the appropriate representation for missing values, such as “0,” “1,” or “NA.” For instance, if zeros signify missing values in your dataset, select “0”.

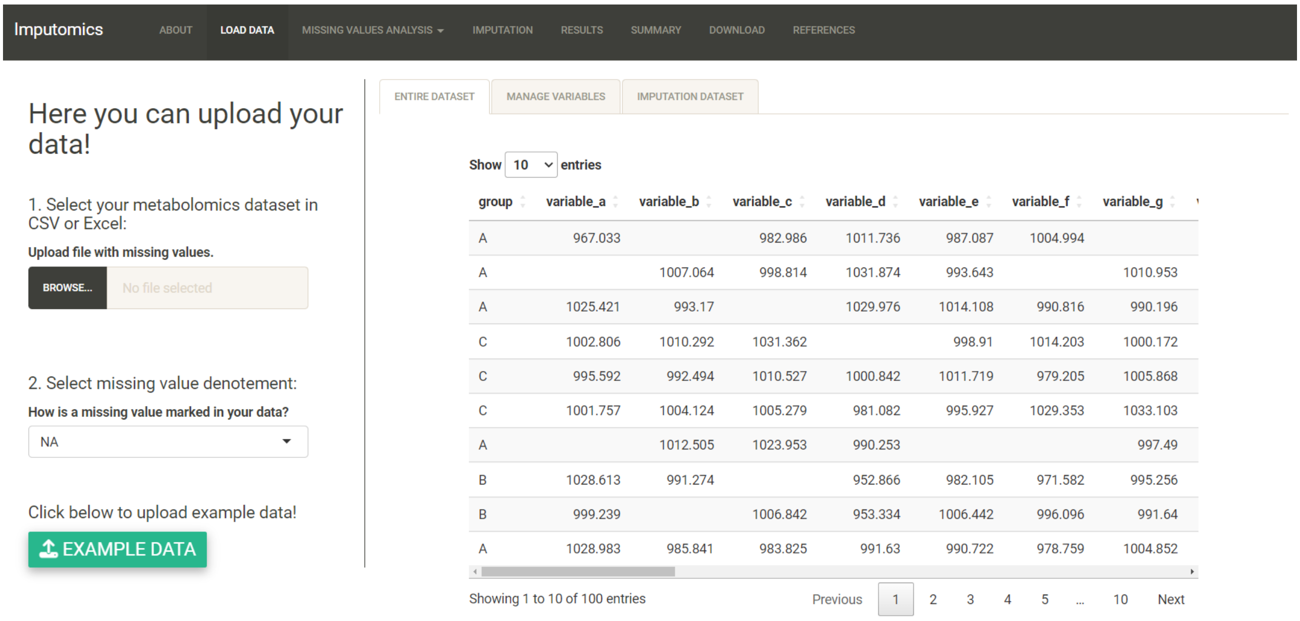

Figure S7: **Uploading data in the *imputomics web server***

Within the “Manage Variables” tab, you are able to specify columns that should be excluded from the imputation process. This involves marking columns that, for instance, represent categorical variables, such as biological groups, but were inadvertently loaded as numerical data.

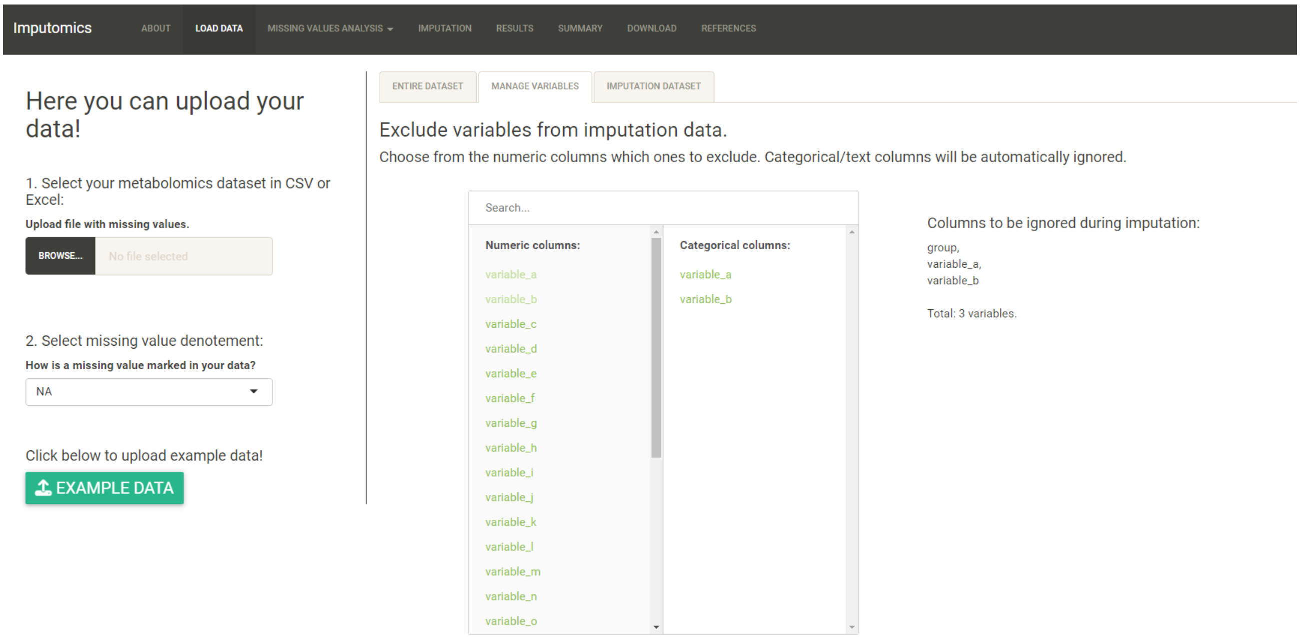

Figure S8: **Managing variables after uploading.**

Within the “Imputation Dataset” tab, you can review the variables that will be included in the imputation process.

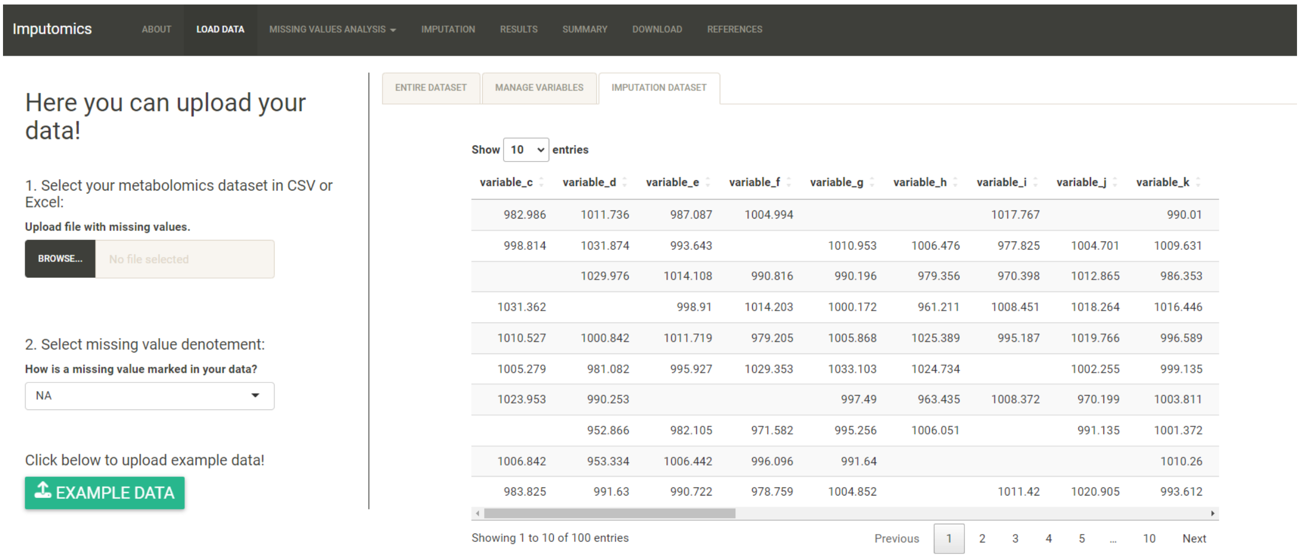

Figure S9: **Imputation dataset preview**

Moving on to the “Visualization” panel, you gain insights into the distribution of missing observations throughout the entire dataset. This includes details such as the percentage of gaps in each column and the overall pattern of missing data. The charts’ appearance can be tailored to your preferences, and you have the option to download them using the “Download” button.

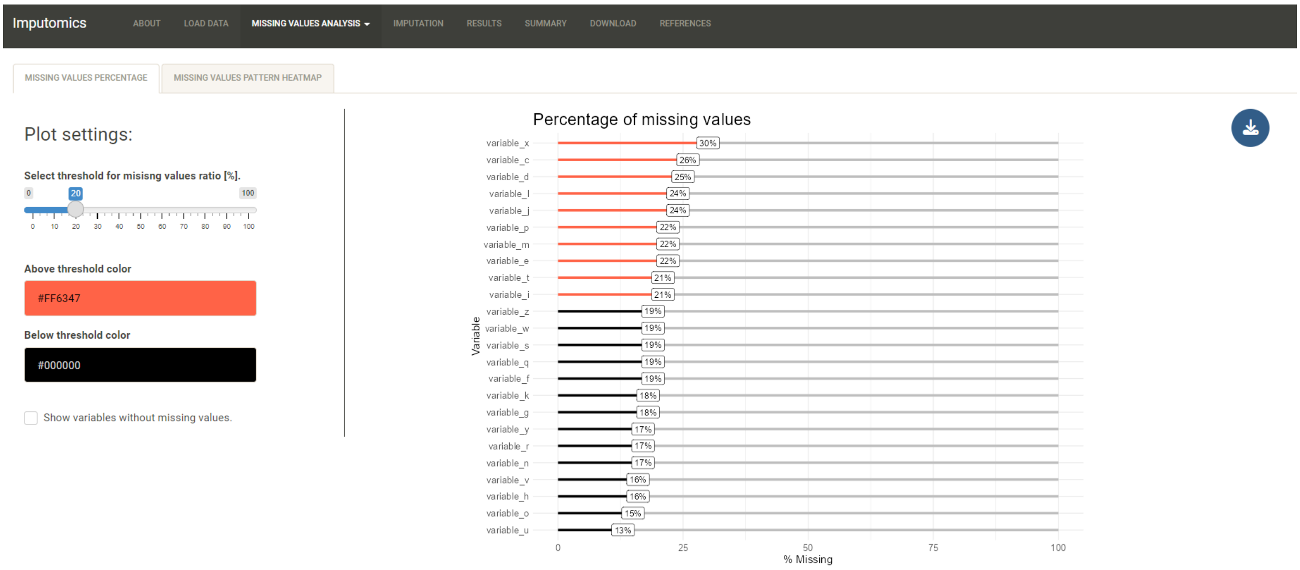

Figure S10: **Visualization of missing values frequency. Percentage occurrence of missing values per each column.**

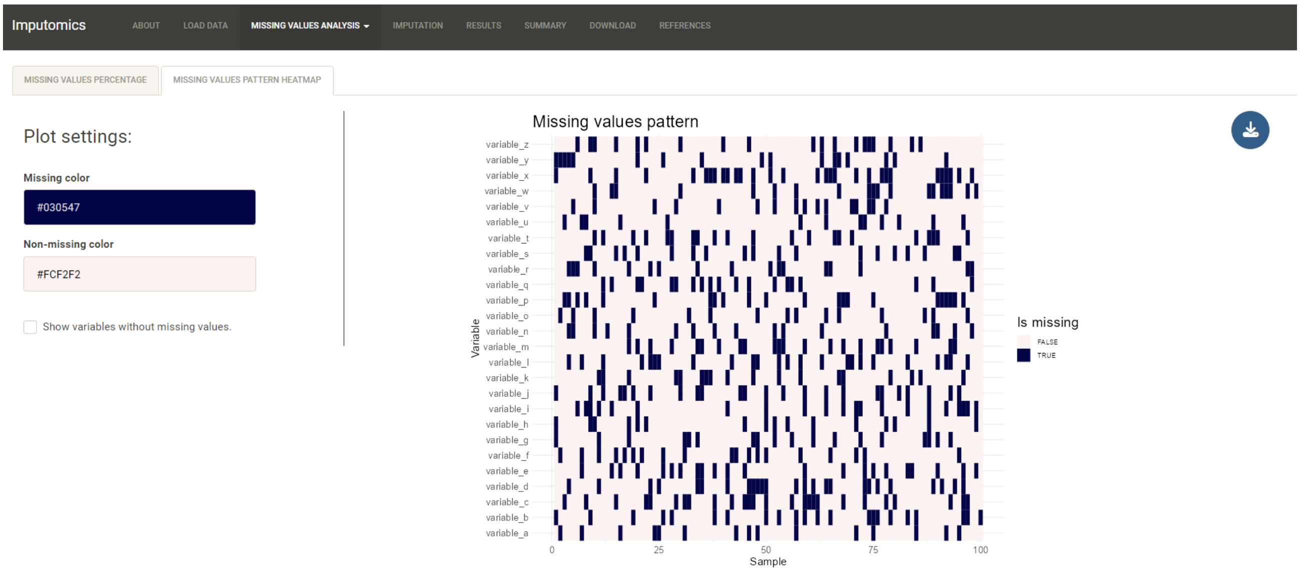

Figure S11: **Visualization of missing values pattern. Heatmap of missing values against the background of entire dataset.**

Subsequently, within the “Missing Values Analysis” tab, you can execute the removal of variables based on the 80% rule, meaning variables with less than 80% observed values will be excluded. Furthermore, when selecting a column describing biological groups, you are able to eliminate variables with less than 80% observations in each group. The application also provides the option to specify a threshold other than 80%.

Following that, in the “Imputation” tab, you can choose the methods you wish to apply for imputing your data. The *imputomics* web server facilitates the utilization of various methods for handling missing values, including but not limited to: amelia, areg, bayesmetab, bcv svd, bpca, cm, corknn, eucknn, halfmin, imputation knn, knn, mean, median, metabimpute bpca, metabimpute halfmin, metabimpute mean, metabimpute median, metabimpute min, metabimpute

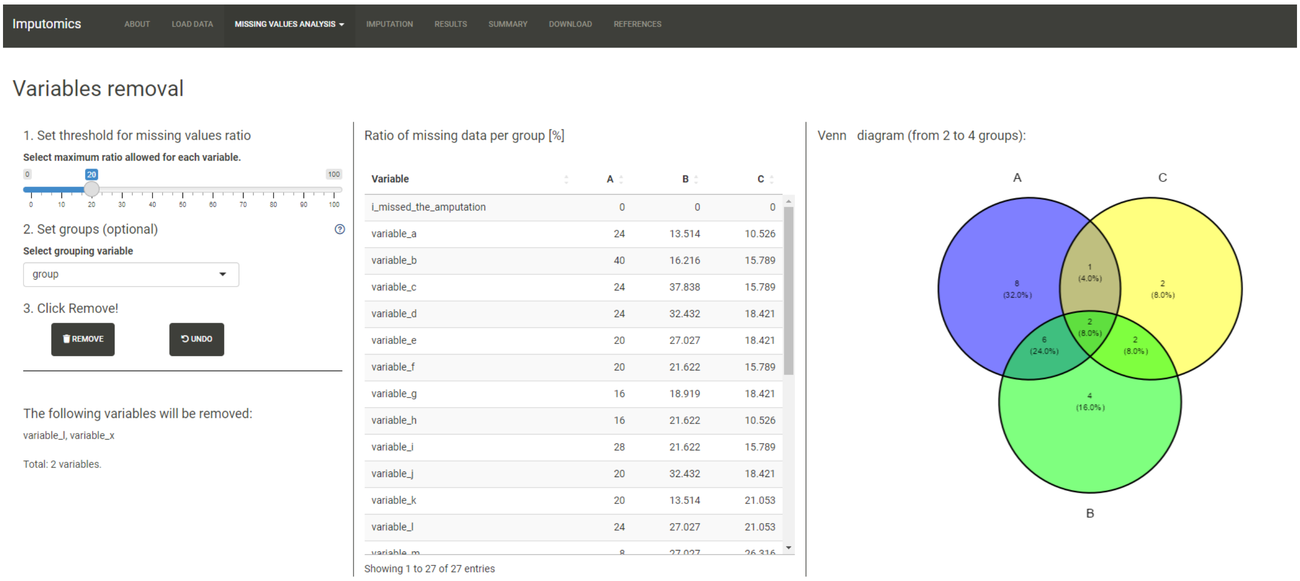

Figure S12: **Visualization of missing values pattern. Heatmap of missing values against the background of entire dataset.**

qrilc, metabimpute rf, metabimpute zero, mice cart, mice pmm, mice rf, min, missforest, missmda em, mnmf, nipals, pemm, ppca, qrilc, random, regimpute, softimpute, svd, tknn, vim knn, zero. Additionally, MAI and Gibbs Sampler based methods turned out to be very resource demanding or unstable and thus are available only in the R package.

Utilizing the switches "Add the 10 fastest methods" and "Add the 10 best-performing methods," you can effortlessly augment the list of selected methods with those that have demonstrated superior speed or performance in terms of NRMSE (Normalized Root Mean Squared Error) and a high frequency of successful imputation in our simulations.

Moreover, you have the option to choose 5 methods that have proven to be the most effective in scenarios involving specific missing value patterns, such as MCAR, MAR and MNAR.

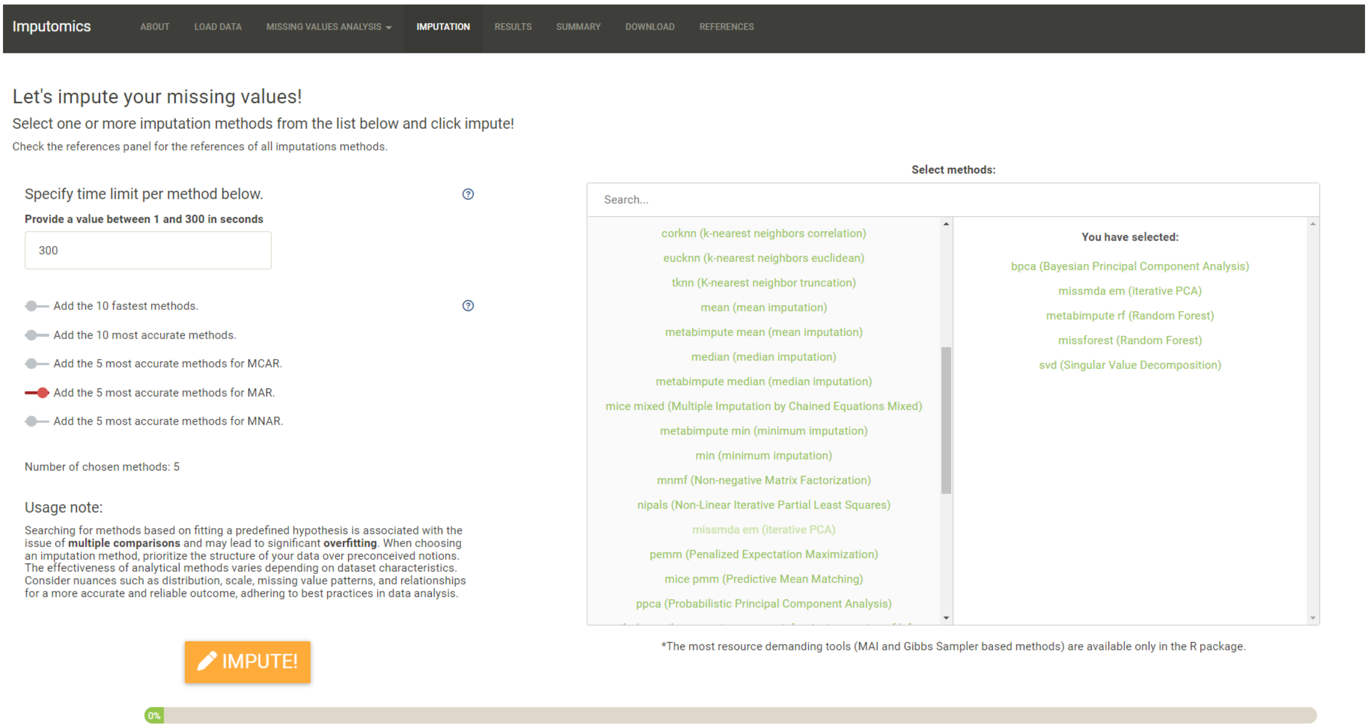

Figure S13: **Missing values imputation.**

In the "Results" tab, you can inspect the data frame post-imputation for each method. Meanwhile, within the "Summary" tab, a chart is available illustrating the distribution of imputed data in contrast to the observed data.

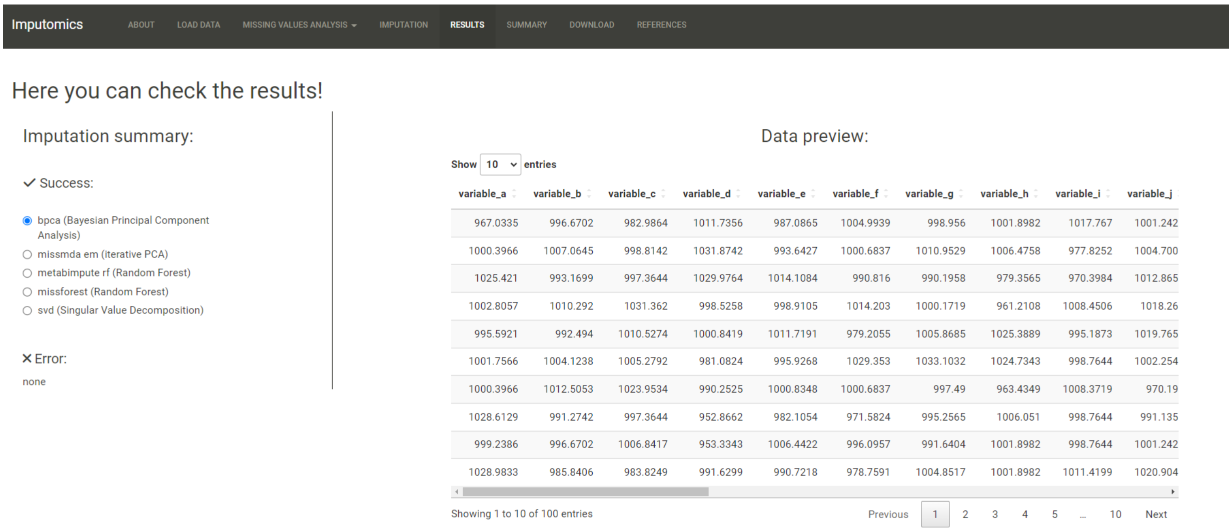

Figure S14: **Results of imputation contained in the table.**

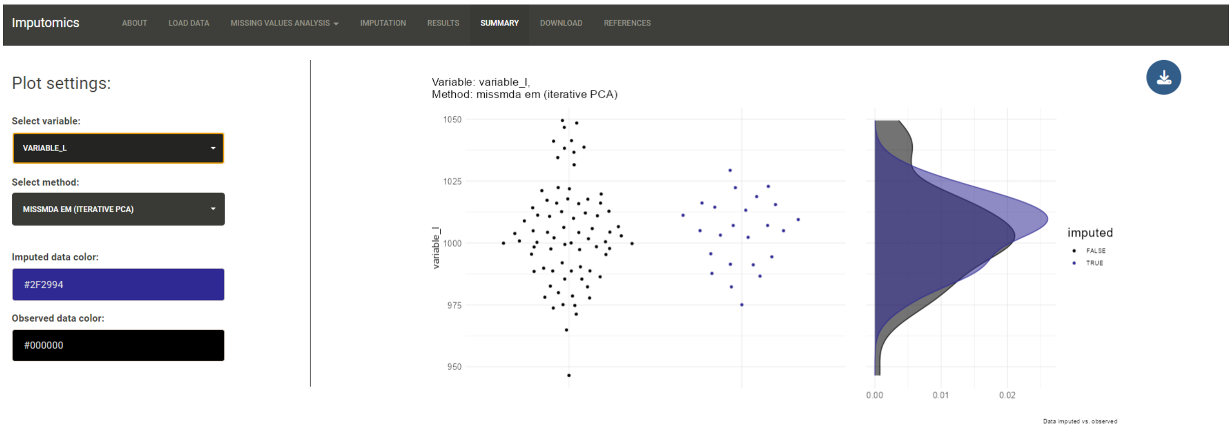

Figure S15: **Visualisation of the results: imputed data againts observed data.**

Finally, within the “Download” tab, you have the option to download an Excel spreadsheet containing the imputation results for each method, presented in individual sheets.

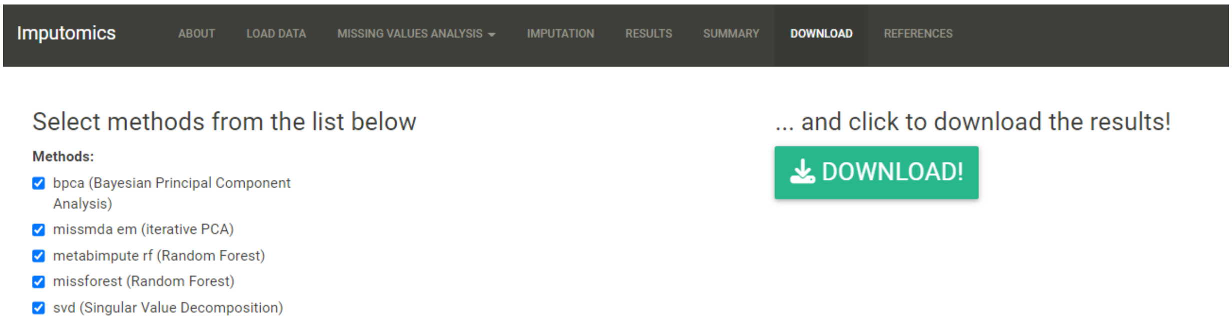

Figure S16: **Downloading the results**

At any time you can go to the References tab to see the references to all the methods used in our package (and web server).

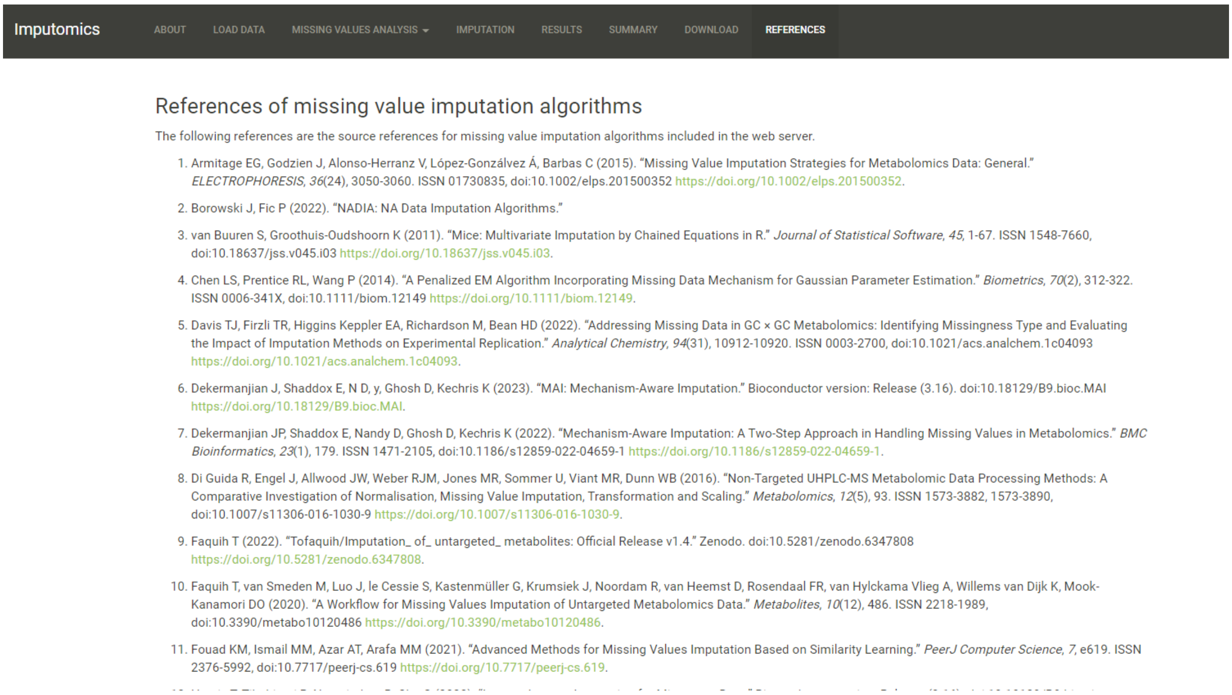

Figure S17: **References.**

SI9 References

Bizzarri, D., M. J. T. Reinders, M. Beekman, P. E. Slagboom, Bbmri-nl, and E. B. van den Akker. 2022. "1H-NMR Metabolomics-Based Surrogates to Impute Common Clinical Risk Factors and Endpoints." *eBioMedicine* 75 (January): 103764. https://doi.org/10.1016/j.ebiom.2021.103764.

Davis, Trenton J., Tarek R. Firzli, Emily A. Higgins Keppler, Matthew Richardson, and Heather D. Bean. 2022. "Addressing Missing Data in GC × GC Metabolomics: Identifying Missingness Type and Evaluating the Impact of Imputation Methods on Experimental Replication." *Analytical Chemistry* 94 (31): 10912–20. https://doi.org/10.1021/acs.analchem.1c04093.

Dekermanjian, Jonathan P., Elin Shaddox, Debmalya Nandy, Debashis Ghosh, and Katerina Kechris. 2022. "Mechanism-Aware Imputation: A Two-Step Approach in Handling Missing Values in Metabolomics." *BMC Bioinformatics* 23 (1): 179. https://doi.org/10.1186/s12859-022-04659-1.

Di Guida, Riccardo, Jasper Engel, J. William Allwood, Ralf J. M. Weber, Martin R. Jones, Ulf Sommer, Mark R. Viant, and Warwick B. Dunn. 2016. "Non-Targeted UHPLC-MS Metabolomic Data Processing Methods: A Comparative Investigation of Normalisation, Missing Value Imputation, Transformation and Scaling." *Metabolomics* 12 (5): 93. https://doi.org/10.1007/s11306-016-1030-9.

Faquih, Tariq, Maarten van Smeden, Jiao Luo, Saskia le Cessie, Gabi Kastenmüller, Jan Krumsiek, Raymond Noordam, et al. 2020. "A Workflow for Missing Values Imputation of Untargeted Metabolomics Data." *Metabolites* 10 (12): 486. https://doi.org/10.3390/metabo10120486.

Jin, Zhuxuan, Jian Kang, and Tianwei Yu. 2018. "Missing Value Imputation for LC-MS Metabolomics Data by Incorporating Metabolic Network and Adduct Ion Relations." Edited by Jonathan Wren. *Bioinformatics* 34 (9): 1555–61. https://doi.org/10.1093/bioinformatics/btx816.

Kokla, Marietta, Jyrki Virtanen, Marjukka Kolehmainen, Jussi Paananen, and Kati Hanhineva. 2019. "Random Forest-Based Imputation Outperforms Other Methods for Imputing LC-MS Metabolomics Data: A Comparative Study." *BMC Bioinformatics* 20 (1): 492. https://doi.org/10.1186/s12859-019-3110-0.

Kumar, Nishith, Md Aminul Hoque, and Masahiro Sugimoto. 2021. "Kernel Weighted Least Square Approach for Imputing Missing Values of Metabolomics Data." *Scientific Reports* 11 (1): 11108. https://doi.org/10.1038/s41598-021-90654-0.

Li, Qian, Kate Fisher, Wenjun Meng, Bin Fang, Eric Welsh, Eric B Haura, John M Koomen, Steven A Eschrich, Brooke L Fridley, and Y Ann Chen. 2020. "GMSimpute: A Generalized Two-Step Lasso Approach to Impute Missing Values in Label-Free Mass Spectrum Analysis." Edited by Jonathan Wren. *Bioinformatics* 36 (1): 257–63. https://doi.org/10.1093/bioinformatics/btz488.

Miller, Hunter A., Ramy Emam, Chip M. Lynch, Samuel Bockhorst, and Hermann B. Frieboes. 2021. "Discrepancies in Metabolomic Biomarker Identification from Patient-Derived Lung Cancer Revealed by Combined Variation in Data Pre-Treatment and Imputation Methods." *Metabolomics* 17 (4): 37. https://doi.org/10.1007/s11306-021-01787-2.

Orešič, Matej, Jussi P. Posti, Maja H. Kamstrup-Nielsen, Riikka S. K. Takala, Hester F. Lingsma, Ismo Mattila, Sirkku Jäntti, et al. 2016. "Human Serum Metabolites Associate With Severity and Patient Outcomes in Traumatic Brain Injury." *EBioMedicine* 12 (July): 118–26. https://doi.org/10.1016/j.ebiom.2016.07.015.

R Core Team. 2022. *R: A Language and Environment for Statistical Computing*. Manual. Vienna, Austria: R Foundation for Statistical Computing.

Shah, Jasmit S., Shesh N. Rai, Andrew P. DeFilippis, Bradford G. Hill, Aruni Bhatnagar, and Guy N. Brock. 2017. "Distribution Based Nearest Neighbor Imputation for Truncated High Dimensional Data with Applications to Pre-Clinical and Clinical Metabolomics Studies." *BMC Bioinformatics* 18 (1): 114. https://doi.org/10.1186/s12859-017-1547-6.

Shah, Jasmit, Guy N. Brock, and Jeremy Gaskins. 2019. "BayesMetab: Treatment of Missing Values in Metabolomic Studies Using a Bayesian Modeling Approach." *BMC Bioinformatics* 20 (S24): 673. https://doi.org/10.1186/s12859-019-3250-2.

Shahjaman, Md., Md. Rezanur Rahman, Tania Islam, Md. Rabiul Auwul, Mohammad Ali Moni, and Md. Nurul Haque Mollah. 2021. "rMisbeta: A Robust Missing Value Imputation Approach in Transcriptomics and Metabolomics Data." *Computers in Biology and Medicine* 138 (November): 104911. https://doi.org/10.1016/j.compbiomed.2021.104911.

Taylor, Sandra L., L. Renee Ruhaak, Karen Kelly, Robert H. Weiss, and Kyoungmi Kim. 2016. "Effects of Imputation on Correlation: Implications for Analysis of Mass Spectrometry Data from Multiple Biological Matrices." *Briefings in Bioinformatics*, February, bbw010. https://doi.org/10.1093/bib/bbw010.

Taylor, Sandra, Matthew Ponzini, Machelle Wilson, and Kyoungmi Kim. 2022. "Comparison of Imputation and Imputation-Free Methods for Statistical Analysis of Mass Spectrometry Data with Missing Data." *Briefings in Bioinformatics* 23 (1): bbab353. https://doi.org/10.1093/bib/bbab353.

Wei, Runmin, Jingye Wang, Erik Jia, Tianlu Chen, Yan Ni, and Wei Jia. 2018a. "GSimp: A Gibbs Sampler Based Left-Censored Missing Value Imputation Approach for Metabolomics Studies." *PLOS Computational Biology* 14 (1): e1005973. https://doi.org/10.1371/journal.pcbi.1005973.

Wei, Runmin, Jingye Wang, Mingming Su, Erik Jia, Shaoqiu Chen, Tianlu Chen, and Yan Ni. 2018. "Missing Value Imputation Approach for Mass Spectrometry-based Metabolomics Data." *Scientific Reports* 8 (1): 663. https://doi.org/10.1038/s41598-017-19120-0.

Wilson, Machelle D., Matthew D. Ponzini, Sandra L. Taylor, and Kyoungmi Kim. 2022. "Imputation of Missing Values

for Multi-Biospecimen Metabolomics Studies: Bias and Effects on Statistical Validity." *Metabolites* 12 (7): 671. https://doi.org/10.3390/metabo12070671.

Xu, Jingjing, Yuanshan Wang, Xiangnan Xu, Kian-Kai Cheng, Daniel Raftery, and Jiyang Dong. 2021. "NMF-Based Approach for Missing Values Imputation of Mass Spectrometry Metabolomics Data." *Molecules* 26 (19): 5787. https://doi.org/10.3390/molecules26195787.
